# Lysophosphatidylcholine Regulates Sexual Stage Differentiation in the Human Malaria Parasite *Plasmodium falciparum*

**DOI:** 10.1016/j.cell.2017.10.020

**Published:** 2017-12-14

**Authors:** Nicolas M.B. Brancucci, Joseph P. Gerdt, ChengQi Wang, Mariana De Niz, Nisha Philip, Swamy R. Adapa, Min Zhang, Eva Hitz, Igor Niederwieser, Sylwia D. Boltryk, Marie-Claude Laffitte, Martha A. Clark, Christof Grüring, Deepali Ravel, Alexandra Blancke Soares, Allison Demas, Selina Bopp, Belén Rubio-Ruiz, Ana Conejo-Garcia, Dyann F. Wirth, Edyta Gendaszewska-Darmach, Manoj T. Duraisingh, John H. Adams, Till S. Voss, Andrew P. Waters, Rays H.Y. Jiang, Jon Clardy, Matthias Marti

**Affiliations:** 1 Wellcome Centre for Molecular Parasitology, Institute of Infection, Immunity & Inflammation, College of Medical, Veterinary and Life Sciences, University of Glasgow, Glasgow G12 8QQ, UK; 2 Harvard T.H. Chan School of Public Health, Department of Immunology and Infectious Diseases, Boston, MA 02155, USA; 3 Harvard Medical School, Department of Biological Chemistry and Molecular Pharmacology, Boston, MA 02155, USA; 4 Center for Global Health & Infectious Diseases Research, Department of Global Health, College of Public Health, University of South Florida, Tampa, FL 33620, USA; 5 Swiss Tropical and Public Health Institute, 4051 Basel, Switzerland; 6 University of Basel, 4001 Basel, Switzerland; 7 Department of Pharmaceutical and Organic Chemistry, Faculty of Pharmacy, University of Granada, 18010 Granada, Spain; 8 Institute of Technical Biochemistry, Faculty of Biotechnology and Food Sciences, Lodz University of Technology, 90-924 Lodz, Poland; 9 Centre for Immunity, Infection and Evolution, Institute for Immunology and Infection Research, University of Edinburgh, Edinburgh EH9 3FL, UK

**Keywords:** *Plasmodium falciparum*, malaria, transmission, sexual differentiation, lysophosphatidylcholine, phospholipid metabolism, environmental sensing, Kennedy pathway

## Abstract

Transmission represents a population bottleneck in the *Plasmodium* life cycle and a key intervention target of ongoing efforts to eradicate malaria. Sexual differentiation is essential for this process, as only sexual parasites, called gametocytes, are infective to the mosquito vector. Gametocyte production rates vary depending on environmental conditions, but external stimuli remain obscure. Here, we show that the host-derived lipid lysophosphatidylcholine (LysoPC) controls *P. falciparum* cell fate by repressing parasite sexual differentiation. We demonstrate that exogenous LysoPC drives biosynthesis of the essential membrane component phosphatidylcholine. LysoPC restriction induces a compensatory response, linking parasite metabolism to the activation of sexual-stage-specific transcription and gametocyte formation. Our results reveal that malaria parasites can sense and process host-derived physiological signals to regulate differentiation. These data close a critical knowledge gap in parasite biology and introduce a major component of the sexual differentiation pathway in *Plasmodium* that may provide new approaches for blocking malaria transmission.

## Highlights

• *P. falciparum* sexual differentiation is repressed by LysoPC found in human serum
• LysoPC is a major building block of parasite phospholipid metabolism
• LysoPC acts upstream of the earliest known events of sexual differentiation
• Parasite metabolism alters host LysoPC levels during infection

## Graphical Abstract

**Figure:**
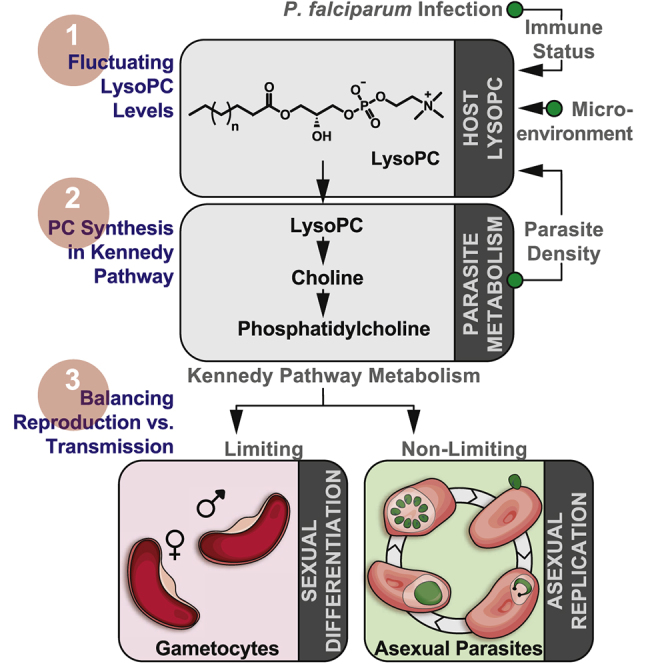

## Teaser

The host-derived lipid lysophosphatidylcholine controls *Plasmodium falciparum* cell fate by repressing parasite sexual differentiation, a key step in malaria transmission.

## Introduction

Malaria remains one of the major global public health threats, with an estimated 200 million clinical cases and 429,000 deaths in 2015 (WHO, 2016). Mature *Plasmodium falciparum* gametocytes are the only stage of the malaria parasite able to establish an infection in the *Anopheles* mosquito vector. Therefore, gametocyte formation and maturation are critical steps for successful parasite dissemination in the human population, and major targets of the ongoing malaria elimination agenda (Nilsson et al., 2015).

Gametocyte densities vary during infection and increase with disease progression, in particular during anemia (Joice et al., 2014, Price et al., 1999). In contrast to asexual stages, the transmissible gametocyte forms are non-proliferative and appear largely quiescent during their development in bone marrow and spleen (Nilsson et al., 2015). Successful parasite transmission therefore requires balancing the investments in asexual reproduction and gametocyte formation under variable host conditions (Reece et al., 2009). Various parameters have been proposed to affect the rate at which gametocytes are produced, including host cell age (Peatey et al., 2013), endoplasmic reticulum (ER) stress (Chaubey et al., 2014), antimalarial treatment (Buckling et al., 1999), and the presence of extracellular vesicles (Mantel et al., 2013, Regev-Rudzki et al., 2013).

On a molecular level, gametocyte formation depends on activation of a stage-specific transcription factor, AP2-G, marking the first known step in sexual differentiation of malaria parasites (Kafsack et al., 2014, Sinha et al., 2014). Sexual differentiation is generally repressed by the co-operative action of histone deacetylase 2 (HDA2) and heterochromatin protein 1 (HP1)—two epigenetic factors required for keeping the *ap2-g* locus in its silenced state (Brancucci et al., 2014, Coleman et al., 2014). Upon expression, AP2-G acts as a transcriptional master switch that induces commitment to the sexual pathway and irreversibly primes the cell for gametocyte development in the following intra-erythrocytic cycle (Kafsack et al., 2014, Sinha et al., 2014). While sexual conversion rates vary across strains and conditions and the transcriptional program activated by AP2-G has been characterized in some detail, no external signals or upstream factors regulating the epigenetic control of *ap2-g* have been identified.

Here we examined how parasite populations regulate entry into the sexual pathway. We used an *in vitro* assay designed to probe the effect of culture perturbations on gametocyte formation (Brancucci et al., 2015, Buchholz et al., 2011) and discovered a serum factor that provides malaria parasites with essential nutrients and controls sexual commitment upstream of AP2-G. Our data reveal the existence of a dynamic crosstalk between *P. falciparum* metabolism and gene regulation and pave the way for systematic dissection of the pathways translating environmental sensing into epigenetic remodeling, *ap2-g* activation, and parasite differentiation.

## Results

### A Soluble Serum Factor Reversibly Represses Sexual Commitment in *P. falciparum*

**Figure 1:**
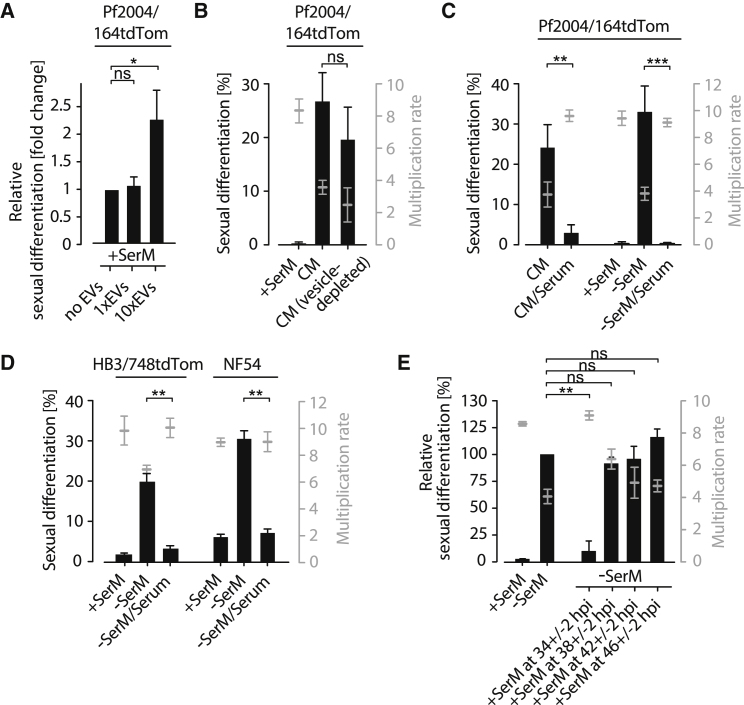
*P. falciparum* Sexual Commitment Is Subject to Host-Factor Availability (A) EVs induce gametocyte formation at high concentrations. Parasites were challenged with EVs isolated from high-parasitemia cultures at the original (1×) and 10× concentrations. Bars show fold change of sexual differentiation normalized to the untreated (no EVs) condition. Absolute differentiation rates were generally low (average of 0.41% in “no EVs” and 1.12% in “10xEVs” condition). n = 3, standard deviations are shown; ns, not significant; ^∗^ p < 0.05, Student’s t test. Parasites of strain Pf2004 were used (transfected with reporter plasmid 164tdTom [Pf2004/164tdTom]). (B) Conditioned medium induces sexual commitment independently of parasite-derived vesicles. +SerM, serum-supplemented medium; CM, conditioned medium. Bars show sexual differentiation rates (left axis). Effect on parasite growth is indicated (right axis). n = 3; standard deviations are shown; ns, not significant, Student’s t test. (C) Parasites induce sexual differentiation in response to serum depletion in −SerM conditions. n = 3, standard deviations are shown, ^∗∗^ p < 0.01, ^∗∗∗^ p < 0.001, Student’s t test. CM, conditioned medium; “/Serum” indicates supplementation with 10% serum. (D) Serum depletion induces sexual commitment across different *P. falciparum* strains. n = 3, standard deviations are shown, ^∗∗^ p<0.01, Student’s t test. Parasites of strain HB3 (transfected with reporter plasmid 748tdTom [HB3/748tdTom]) and wild-type parasites of strain NF54 were used. (E) Sexual differentiation is irreversibly determined after 38 ± 2 hpi. Except for the +SerM control, all cultures were exposed to −SerM at 28 ± 2 hpi. −SerM was exchanged by +SerM at indicated time points. n = 3, standard deviations are shown, ^∗∗^ p < 0.01; ns, not significant; Student’s t test.

Exposing parasites to spent or “parasite-conditioned” medium (CM) dramatically induces sexual commitment relative to fresh medium complemented with human serum (+SerM) (Brancucci et al., 2015, Buchholz et al., 2011, Williams, 1999). Based on these data, it was hypothesized that CM was either enriched in specific secreted parasite factors or depleted of specific host components. In favor of the former hypothesis, we have recently demonstrated that extracellular vesicles (EVs) from CM can increase sexual commitment rates (Mantel et al., 2013). Further experiments confirmed that EVs can induce parasite differentiation when applied at high concentrations (Figure 1A). However, CM retains most of its activity after EVs have been depleted from the medium (Figure 1B), suggesting that vesicles are not essential for gametocyte formation. Conversely, we found that the addition of serum is sufficient to reverse the commitment-inducing effect of CM. Consistent with a model in which gametocyte formation is triggered in response to restricted host factor availability, sexual commitment could be induced by exposing parasites to a serum-free medium (−SerM) (Figure 1C). In −SerM, human serum is substituted by bovine serum albumin and a minimal set of fatty acids required for parasite survival (Mi-Ichi et al., 2007, Mitamura et al., 2000). Together, these data support the hypothesis that components of human serum prevent sexual differentiation and that these components are depleted in CM. −SerM culture conditions enhance gametocyte production across strains (Figure 1D), demonstrating that sexual commitment is a conserved response of *P. falciparum* parasites to serum depletion. Whereas the commitment-inducing effect of −SerM conditions could be reversed by the addition of serum up to 34 ± 2 hr post invasion (hpi), rescue attempts at 38 ± 2 hpi and later time points were ineffectual (Figure 1E), indicating that sexual differentiation is irreversibly determined between 34 ± 2 and 38 ± 2 hpi.

### Identification of Lysophosphatidylcholine as the Active Serum Component Blocking Sexual Commitment

**Figure 2:**
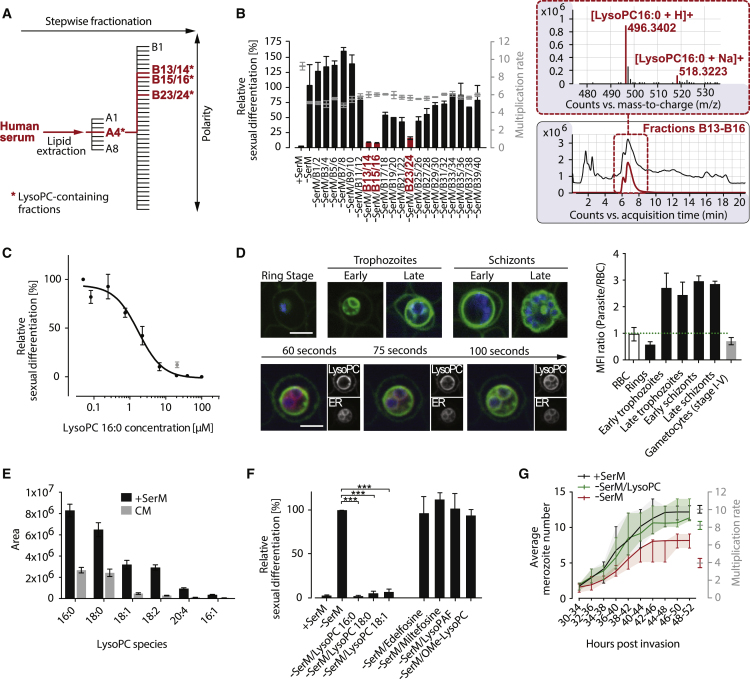
Identification of LysoPC as a Regulator of Transmission-Stage Formation in *P. falciparum* (A) Polarity-based separation of serum components yields LysoPC-enriched fractions with sexual differentiation-inhibiting activity (highlighted in red). See Figure S1 for details on activity and composition of fractions. (B) Sexual commitment inhibiting activity of LysoPC-containing fractions (left panel) and LC-MS chromatograms and mass spectra of most active fractions (right panel) are shown. An extracted ion chromatogram for the [M+H]^+^ ion of LysoPC (16:0) is highlighted in red. Bars in the left panel quantify sexual differentiation normalized to a −SerM-exposed control population. n = 3, data from a representative experiment is shown. Standard deviations of technical triplicates are indicated. (C) LysoPC inhibits sexual differentiation of Pf2004/164tdTom parasites (half maximal inhibitory concentration [IC50]). Effect of 20 μM LysoPC on HB3/748tdTom parasites is shown in grey. Sexual differentiation is normalized to −SerM-cultured control populations. n = 3, standard errors are shown. (D) LysoPC is internalized by asexual *P. falciparum* parasites. Uptake was analyzed by live microscopy using TopFluor-labeled LysoPC (green), ER tracker (red) and DNA dye Hoechst (blue). Incorporation is apparent after completion of ring stage development (upper left panel) and the label accumulates at the ER of trophozoites within 75 s after addition of TopFluor LysoPC (lower left panel). LysoPC uptake in the parasite is quantified relative to accumulation at the erythrocyte surface (right panel). Mean fluorescence intensities (MFIs) and standard deviations are shown. Scale bar, 4 μm. 100 cells were analyzed per stage in triplicate experiments. (E) LysoPC is depleted in parasite-exposed CM. Bars quantify area under the curve. n = 3, standard errors are shown. (F) In contrast to different LysoPC species, non-hydrolyzable analogs of LysoPC fail to prevent −SerM-induced sexual differentiation. All molecules were tested at 20 μM. n = 3, standard deviations are shown, ^∗∗∗^ p < 0.001, Student’s t test. See Figure S1C for chemical structures. (G) LysoPC-depleted culture conditions (−SerM) result in reduced number of daughter merozoites per schizont (left axis) and reduced multiplication rate (right axis) compared to +SerM and −SerM/LysoPC conditions. n = 100. Interquartile ranges are shown. See also Figures S1 and S2.

**Figure S1:**
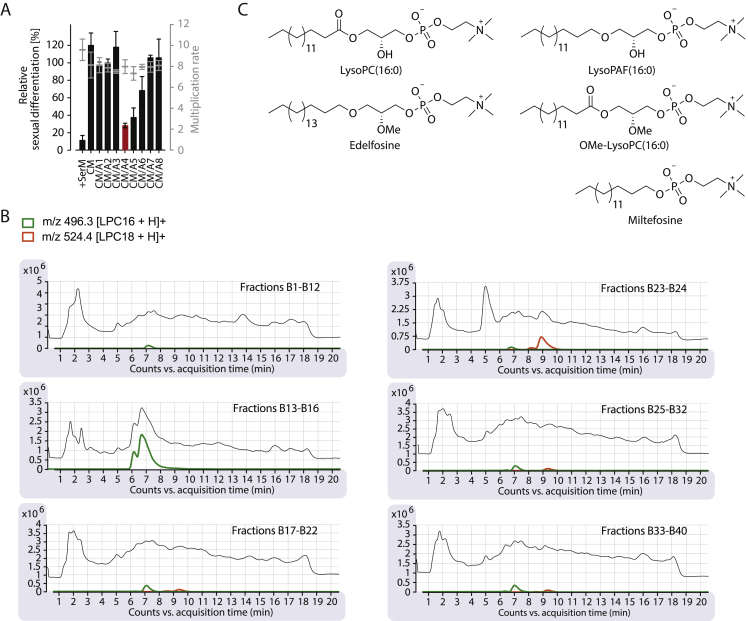
Related to Figure 2 (A) Stepwise fractionation of serum (fractions A1–A8) identifies LysoPC as the active component. See Figure 2A for schematic of experimental approach. Bars quantify sexual differentiation normalized to a −SerM- or CM-exposed control population. Active fractions were tested in biological triplicates or quadruplicates as described (Brancucci et al., 2015). Data from a representative experiment is shown and standard deviations of technical triplicates are indicated. CM, 80% conditioned medium. (B) LC-MS chromatograms and mass spectra of B fractions (see above) are shown; LysoPC species are highlighted in color. (C) Chemical structures of tested analogs.

To identify the molecule(s) present in human serum that control parasite differentiation, we separated its components based on polarity (Figure 2A). The resulting serum fractions were tested for their ability to repress parasite sexual commitment. After two iterative rounds of fractionation and biological assays, several fractions with significant commitment-repressing activity were identified (Figures 2B and S1A). Mass spectrometry analysis revealed lysophosphatidylcholine (LysoPC) species to be major components in these fractions (Figures 2B, S1B, and S1C). Indeed, addition of LysoPC (16:0) efficiently compensated for the absence of serum in −SerM conditions and prevented parasite sexual commitment at a 50% inhibitory concentration of 1.73 μM (95% CI 1.13–2.65 μM) (Figure 2C).

**Figure S2:**
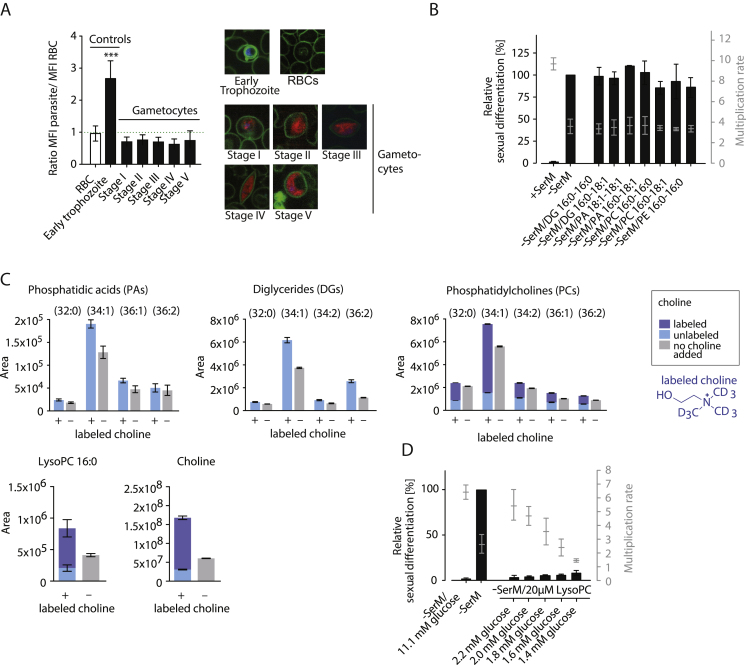
Related to Figures 2 and 3 (A) Live cell microscopy comparing uptake of TopFluor-labeled LysoPC between *P. falciparum* gametocytes and asexual parasites. LysoPC uptake in the parasite is quantified relative to accumulation at the erythrocyte surface (left panel). 100 cells were analyzed per stage. Representative pictures are shown (right panel). Standard deviations are shown, (^∗∗∗^p < 0.001, Student’s t test). (B) Increase of exogenous supply of Kennedy pathway metabolites does not affect *P. falciparum* parasite sexual differentiation. DG, diglycerides; PA, phosphatidic acids; PC, phosphatidylcholines; PE, phosphatidylethanolamine. n = 3, standard deviations are shown. (C) Levels of parasite phosphatidic acids (PAs), diglycerides (DGs), phosphatidylcholines (PCs), LysoPC(16:0) and choline are significantly increased in the presence of a high concentration of choline. Bars show chromatographic areas under the curve. Colors indicate the contribution of labeled and unlabeled molecules to the total peak areas. n = 3, standard errors of the means are shown. (D) Glucose levels have no autonomous effect on parasite sexual differentiation. While glucose can prevent sexual differentiation in absence of LysoPC (see Figure 3F), limiting levels of this sugar do not induce gametocyte formation under LysoPC-rich conditions. n = 3, standard deviations are shown.

Using a fluorescent analog of the lipid, we demonstrated that trophozoites and schizonts, but not younger stage asexual parasites or gametocytes, rapidly incorporated LysoPC or metabolites thereof into parasite membranes (Figures 2D and S2A). These results are suggestive of LysoPC uptake through a parasite-induced channel that is only active in mature asexual stages, such as the recently characterized plasmodial surface anion channel (PSAC) (Nguitragool et al., 2011). In support of active uptake, we found that all LysoPC species detectable by mass spectrometry are significantly depleted in CM compared to fresh serum-complemented medium (Figure 2E). To determine a possible function for serum LysoPC in receptor-mediated signaling, as described in various eukaryotic systems (Gómez-Muñoz et al., 1999, Yang et al., 2005), we tested a series of non-hydrolysable LysoPC analogs (Danker et al., 2010). None of these analogs, including the previously characterized bioactive compounds miltefosine and edelfosine, could block sexual commitment (Figures 2F and S1C). Notably, LysoPC depletion resulted in reduced parasite progeny numbers (Figure 2G). Together, these observations suggested a metabolic link rather than direct receptor-mediated signaling events in LysoPC-mediated regulation of sexual commitment.

### LysoPC Metabolism via the Parasite’s Kennedy Pathway Is Required to Block Sexual Commitment

**Figure 3:**
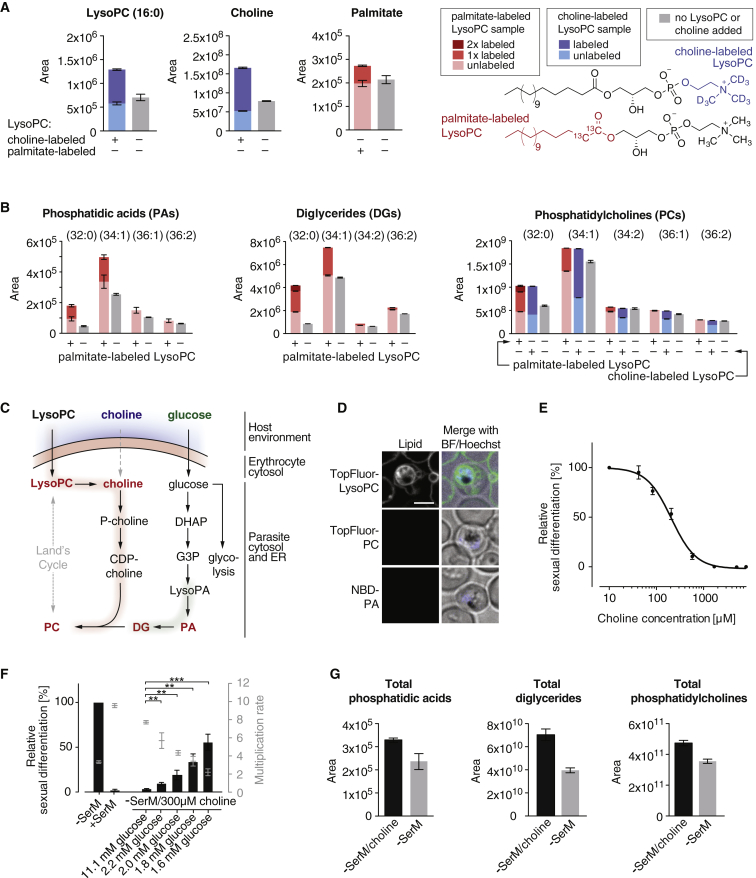
LysoPC Is Metabolized and Drives PC Biosynthesis via the Kennedy Pathway (A) Addition of heavy-isotope-labeled LysoPC increases levels of palmitate and choline in parasites. Bars show chromatographic areas under the curve. Colors indicate the contribution of labeled and unlabeled molecules to the total peak areas. n = 3, standard errors of the means are shown. ^13^C palmitate LysoPC or ^2^H choline LysoPC was used. (B) Levels of parasite phosphatidic acids (PAs), diglycerides (DGs), and phosphatidylcholines (PCs) are significantly increased in presence of LysoPC and elevated lipids contain LysoPC-derived building blocks. Bars show chromatographic areas under the curve. Colors indicate the contribution of labeled and unlabeled molecules to the total peak areas. n = 3, standard errors of the means are shown. (C) The Kennedy pathway requires CDP-choline and DGs deriving from LysoPC and glycolysis products, respectively, for PC synthesis. LysoPA, lysophosphatidic acid; DHAP, dihydroxyacetone phosphate; G3P, glycerol-3-phosphate; PA, phosphatidic acid; P-choline, phosphocholine; CDP-choline, cytidine diphosphate choline; DG, diglyceride. (D) Live cell microscopy shows inefficient incorporation of fluorescent Kennedy metabolites PC (TopFluor-labeled) and PA (nitrobenzoxadiazole [NBD]-labeled) compared to LysoPC (TopFluor-labeled). Scale bar, 4 μm. Representative pictures are shown. (E) Choline inhibits sexual differentiation at super-physiological concentrations (IC50 of 207 μM; 95% CI 169–253 μM); human serum contains ∼10 μM choline (Psychogios et al., 2011). Activity of choline was assayed in presence of 11.1 mM glucose in −SerM medium. n = 3, standard errors are shown. (F) In absence of exogenous LysoPC, glucose is required in addition to excess choline to inhibit sexual differentiation, reiterating the importance of Kennedy-mediated PC synthesis in this process. n = 3, standard deviations are shown, ^∗∗^ p < 0.01, ^∗∗∗^ p < 0.001, Student’s t test. (G) In presence of glucose, exogenously added choline mirrors the effect of LysoPC and elevates levels of parasite PA, DG, and PC. n = 3, standard errors of the means are shown. See also Figure S2 and Dataset S1.

To investigate the role of LysoPC metabolism in sexual commitment, we performed a series of heavy isotope-based metabolic labeling assays. Following a 12-hr incubation period with ^13^C palmitate- or ^2^H choline-labeled LysoPC, parasites were subjected to extraction and liquid chromatography-mass spectrometry (LC-MS) analysis. These experiments revealed that parasites indeed metabolized LysoPC and used it to synthesize choline- and fatty-acid-containing products. The majority of free choline in the parasite (68%) was labeled even in the presence of equimolar amounts of unlabeled choline (21.5 μM) in the medium, suggesting that LysoPC is the major choline source for *P. falciparum* blood stage parasites (Figure 3A and Dataset S1). Relative metabolite quantification further demonstrated that the levels of phosphatidic acids (PAs), diglycerides (DGs), and some species of phosphatidylcholine (PCs) dropped significantly under LysoPC-depleted conditions (Figure 3B and Dataset S1). PCs are the most abundant membrane lipids in *P. falciparum* (Gulati et al., 2015), and PAs and DGs are their direct metabolic precursors in the Kennedy pathway (Figure 3C).

To confirm a direct link between sexual differentiation and the levels of PC (or its precursors, PA and DG), we attempted to block sexual commitment by addition of these lipids to the growth medium. However, parasites failed to efficiently access exogenous pools of these molecules (Figure 3D), explaining their inability to repress sexual commitment (Figure S2B). Next, we altered the parasites’ access to Kennedy pathway substrates choline and glucose in absence of LysoPC. Glucose is metabolized into glycerol-3-phosphate, which in turn is acylated to yield PA and DG; choline is activated into cytidine diphosphate (CDP)-choline before reacting with glucose-derived DG to yield PC (Figure 3C). Therefore, if PC levels regulate sexual commitment, we expected that increasing choline levels block sexual commitment in the presence of sufficient glycolysis by elevating cellular PC via the Kennedy pathway. Indeed, addition of super-physiological concentrations of choline to −SerM medium prevented sexual differentiation (Figure 3E), and this effect was dependent on glucose levels (Figure 3F). Metabolomic analysis confirmed that, similar to LysoPC, the added choline was incorporated into PC and significantly increased its cellular concentration under standard glucose levels (Figures 3G and S2C and Dataset S1). In contrast to limiting levels of LysoPC and choline, glucose depletion is not sufficient to induce commitment independently (Figure S2D).

### LysoPC Depletion Defines the Transcriptional Signature of Sexual Commitment

**Figure 4:**
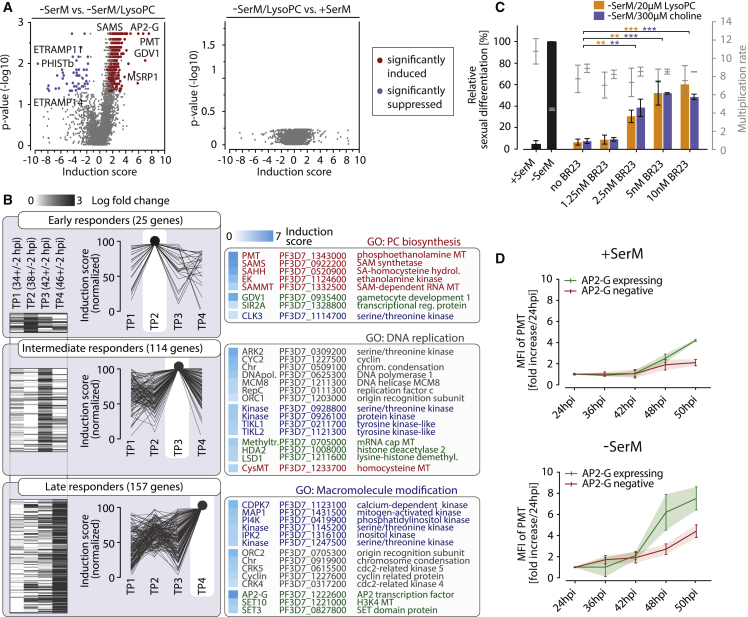
LysoPC Depletion Induces Activity of More Than 300 Genes in *P. falciparum* (A) Transcriptional responses of two parasite strains (Pf2004/164tdTom and NF54; combined) to absence of LysoPC are shown (left panel). Significantly up- and downregulated genes are highlighted in red and blue, respectively. Data show differential transcription between parasites cultured in −SerM and −SerM/20 μM LysoPC. No significant differential expression could be detected between parasites cultured in +SerM and −SerM/20 μM LysoPC (right panel). (B) Clustering of differentially expressed genes reveals that LysoPC depletion elicits a temporal transcriptional cascade in *P. falciparum*. Differential induction of genes (−SerM *vs*. −SerM/LysoPC conditions) per time point is shown in a heat map (left panel) and stacked line graph (middle panel). For each time point, the highest-enriched gene ontology (GO) term (p < 0.05) and a subset of significantly induced genes are shown (right panel). Factors involved in epigenetic regulation and/or differentiation are marked in green. (C) CK inhibitor BR23 (Serrán-Aguilera et al., 2016) reverses activity of LysoPC and excess choline, corroborating importance of the Kennedy pathway in regulating sexual differentiation. n = 3, standard deviations are shown, ^∗∗^ p < 0.01, ^∗∗∗^ p < 0.001, Student’s t test. (D) PMT expression is increased in sexually committed schizonts. Enzyme levels were quantified in a time-course experiment. While PMT is generally induced under −SerM conditions, sexually committed cells show a more prominent induction. MFI, mean fluorescence intensity; a minimum of 100 infected red blood cells (iRBCs) were analyzed by confocal microscopy. Measurements were repeated in triplicate experiments. Stadard errors of the means are shown. See also Figures S3 and S4 and Dataset S2.

**Figure S3:**
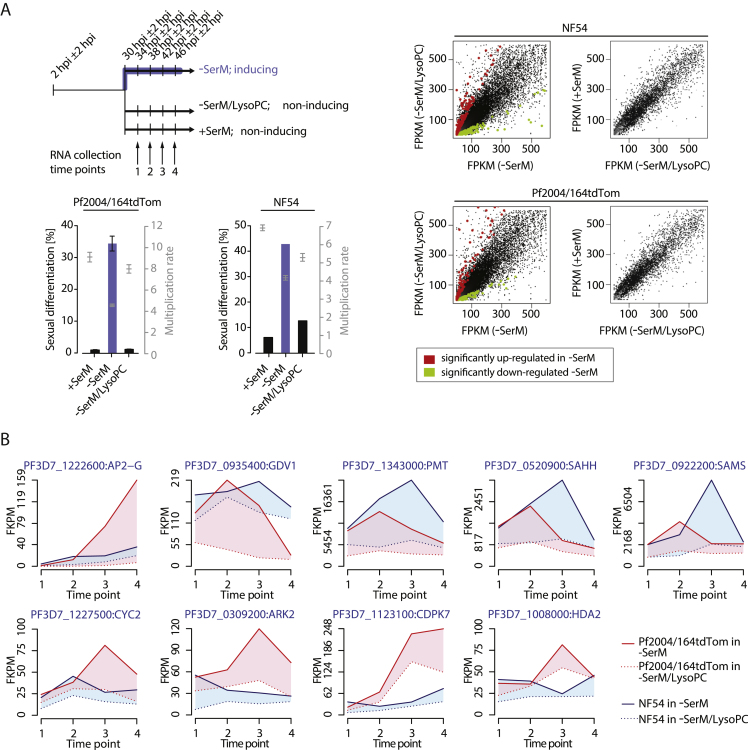
Related to Figure 4 (A) Schematic depiction of parasite culturing setup used for transcriptional analysis (upper left panel). Pf2004/164tdTom and NF54 parasite cultures were split at 30±2 hpi and parasites were subsequently grown under conditions that either induce (−SerM) or do not induce (−SerM/LysoPC; +SerM) sexual differentiation. Time points of RNA harvest are indicated. Effect of culture conditions on parasite sexual differentiation and multiplication is shown (lower left panels, standard deviations are indicated). Global effect of culture conditions on differential gene expression (Pf2004/164tdTom and NF54, combined) is shown in scatterplots on the right. FPKM, fragments per kilobase of exon per million mapped reads. (B) Effect of LysoPC on gene expression of selected genes. Normalized read counts are given for different conditions (−SerM and −SerM/LysoPC) and time points. Differential gene expression is indicated by shaded areas (red, Pf2004/164tdTom; blue, NF54). FPKM, fragments per kilobase of exon per million mapped reads.

To determine the impact of LysoPC depletion on gene expression in the parasite, we performed an RNA-sequencing (RNA-seq) timecourse experiment (Figure S3A). In brief, highly synchronous parasite populations were split at 30 ± 2 hpi and subsequently cultured in either LysoPC-free −SerM or −SerM complemented with 20 μM LysoPC. Parasites grown in +SerM served as a control for conditions that inhibit gametocytogenesis. RNA was then collected in 4-hr increments to investigate gene regulation during sexual commitment. Comparative transcriptional profiling of two *P. falciparum* strains revealed a dramatic induction of *ap2-g* transcription in absence of LysoPC, demonstrating that the lipid acts upstream of the earliest known regulator of differentiation. We found a total of 342 genes significantly induced in response to LysoPC depletion, while 45 genes were downregulated (Figures 4A and S3B and Dataset S2). No significant differences were detected between parasites cultured in serum- and LysoPC-complemented medium (Figure 4A), demonstrating that LysoPC maintains default transcription of blood-stage parasites in an autonomous manner. On the other hand, LysoPC depletion resulted in a cascade effect with a first set of 25 genes induced rapidly after culture conditions were changed (Figure 4B). The prominent induction of metabolism-related genes found within this group, including ethanolamine kinase (*ek*), phosphoethanolamine N-methyltransferase (*pmt*), and genes encoding enzymes required for methyl-donor synthesis (S-adenosylmethionine synthase, SAMS; S-adenosylmethionine methyltransferase, SAMMT), suggests a compensatory utilization of ethanolamine as a Kennedy pathway substrate for PC biosynthesis. Under LysoPC-limiting conditions, elevated *ek* and *pmt* activity may act in concert to catalyze production of the Kennedy substrate phosphocholine (P-choline) from ethanolamine via stepwise phosphorylation and SAM-dependent methylation events, ultimately securing cellular PC levels. It has previously been demonstrated that this alternative Kennedy pathway route is indeed active in blood-stage *P. falciparum* (Pessi et al., 2004).

**Figure S4:**
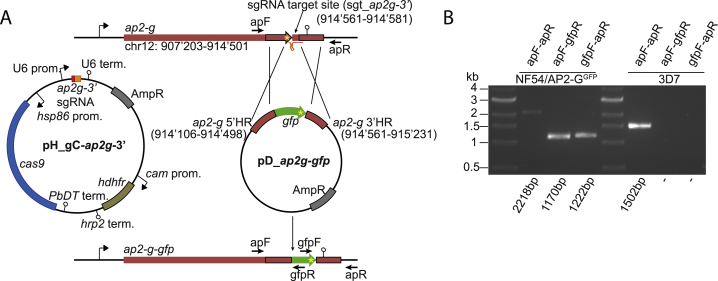
Related to Figure 4 (A) Schematic maps of the endogenous *ap2-g* locus (PF3D7_1222600) in wild-type parasites (top), the pH_gC-*ap2g-3’* and pD_*ap2g-gfp* transfection vectors (center), and the edited *ap2-g-gfp* locus after CRISPR/Cas9-based marker-free fusion of the *gfp* sequence to the 3’end of the *ap2-g* coding sequence in NF54/AP2-G^GFP^ parasites (bottom). Numbers refer to nucleotide positions on chromosome 12 (http://plasmodb.org/). The position of the sgt_*ap2g3’* sgRNA target sequence 60bp downstream of the *ap2-g* coding sequence is indicated. The pH_gC-*ap2g-3’* Cas9/sgRNA suicide plasmid contains expression cassettes for SpCas9, the sgRNA and the h*dhfr* resistance marker. In the pD_*ap2g-gfp* donor plasmid two homology regions (5’HR and 3’HR) flanking the *gfp* coding sequence facilitate repair of the Cas9-induced double-strand break and marker-free tagging of the *ap2-g* gene by double crossover homologous recombination. Yellow asterisks indicate the position of translation termination codons. Positions of PCR primer binding sites used to confirm successful gene editing are indicated by horizontal black arrows. (B) PCR on gDNA isolated from NF54/AP2-G^GFP^ and 3D7 wild-type control parasites. Primers apF and apR bind to wild-type sequences outside the *ap2-g* 5’HR and *ap2-g* 3’HR homology regions and amplify a 2218bp or 1502bp fragment from the edited *ap2-g-gfp* or wild-type *ap2-g* locus, respectively. The ap2F/gfpR and gfpF/ap2R primer combinations are specific for the edited *ap2-g-gfp* locus in NF54/AP2-G^GFP^ parasites and amplify 1170 and 1222bp fragments, respectively.

To evaluate the metabolic changes observed by RNA-seq, we also determined the role of key components of the Kennedy pathway on a protein level. First, we found that specific inhibition of the Kennedy pathway enzyme choline kinase (CK) induces sexual commitment even in the presence of LysoPC or excess choline and glucose (Figure 4C). This result provides direct evidence that biosynthesis of PC species through the Kennedy pathway plays an essential role in suppressing parasite sexual differentiation. LysoPC acylation via the Lands cycle was not assessed in this study but likely contributes to parasite PC production (Déchamps et al., 2010). Second, we investigated the expression of the most highly induced metabolic enzyme, PMT, in single cells in response to LysoPC depletion. PMT catalyzes the methylation events required to produce P-choline from the alternative substrate ethanolamine and thus likely facilitates adaptation to LysoPC-depleted conditions. We generated an endogenously tagged AP2-G reporter line (NF54/AP2-G^GFP^) in order to mark sexually committed schizonts (Figure S4) and used this line to quantify PMT expression by immunofluorescence. Confirming the RNA-seq data, imaging flow cytometry revealed that PMT enzyme levels are elevated in all LysoPC restricted cells. However, compared to asexual parasites, the enzyme reaches significantly higher expression levels in sexually committed schizonts (i.e., AP2-G expressing cells) (Figure 4D). These results further corroborate the existence of an intimate link between parasite metabolism and sexual differentiation and suggest that sexually and asexually committed cells utilize distinct metabolic strategies under LysoPC limiting conditions.

The events that follow this initial metabolic response are dominated by a stepwise induction of epigenetic regulators, kinases, and factors involved in cell-cycle regulation and differentiation (Figure 4B). The presented transcriptional signature of LysoPC depletion will allow for systematic dissection of factors and pathways linking environmental sensing to cellular responses and sexual differentiation.

### Loss of LysoPC-Mediated Regulation of Sexual Commitment in the Rodent Malaria Lineage

**Figure 5:**
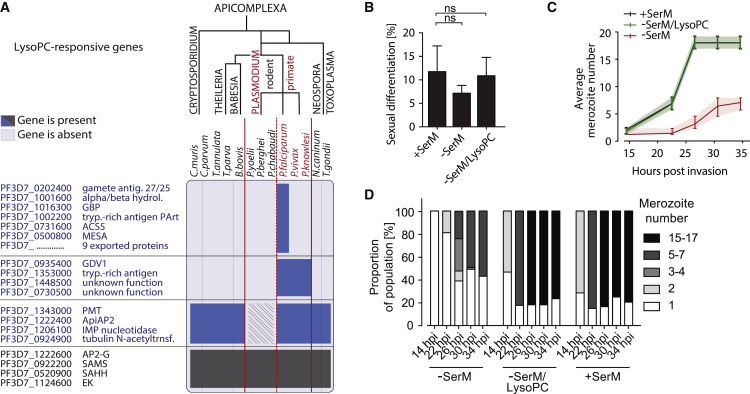
LysoPC Is Metabolized, but Does Not Control Differentiation, in Rodent Malaria Parasites (A) Phylogenetic analysis reveals loss of key LysoPC-responsive genes in the rodent malaria lineage. Lineage-specific losses are marked by hatched area. (B) LysoPC does not affect sexual differentiation of rodent parasites. Gametocyte production of *P. berghei* parasites in response to different *ex vivo* culture conditions (+SerM, −SerM, or −SerM/LysoPC) is shown. Effect on sexual differentiation was measured after parasites were delivered into a recipient mouse. n = 5, standard deviations are shown, (ns, not significant; Student’s t test). (C and D) LysoPC depletion reduces number of progeny in *P. berghei*. Growth response to different *ex vivo* culture conditions (+SerM, −SerM, or −SerM/LysoPC) is shown. (C) Average merozoite counts per infected erythrocyte were quantified after subtracting gametocyte-infected cells (single nucleated cells in +SerM condition at 30 hpi were used to define gametocyte proportion in samples). Interquartile ranges are shown. (D) Merozoite number was quantified per infected erythrocyte and assigned to one of five categories (indicated). Shown are relative proportions of each category. Merozoites of 30–65 infected erythrocytes were counted per time point.

Our data demonstrate that LysoPC depletion induces sexual-stage-specific transcription and gametocyte formation across *P. falciparum* strains. We noted that a subset of LysoPC-responsive genes is specifically absent in the rodent parasite lineage (Figure 5A). This lineage-specific difference may reflect alternative strategies in environmental sensing and sexual differentiation between rodent and primate malaria parasites, including *P. falciparum*. Indeed, we found that gametocyte production by the rodent parasite *P. berghei* remained unaffected by depletion of LysoPC (Figure 5B). However, LysoPC is essential for normal progression of the rodent parasite *P. berghei* through schizogony (Figures 5C and 5D). These results indicate that although *P. berghei* requires LysoPC for proliferation, it regulates sexual differentiation independently from LysoPC-mediated metabolic cues. These parasites must have developed a separate strategy to control transmission stage formation. Notably, *P. berghei* parasites appear to have sexual conversion rates that are generally higher than those of *P. falciparum*, and they can commit (i.e., induce sexual differentiation) and form gametocytes within the same cell cycle (Carter et al., 2013, Mons, 1986). Together, these observations suggest fundamental differences in the regulation of sexual commitment between the two lineages.

### Physiological LysoPC Levels during Malaria Infection Are Conducive to Sexual Commitment

**Figure 6:**
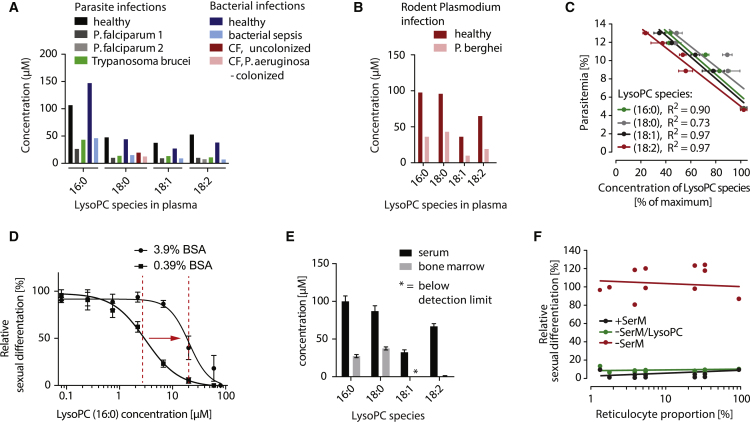
Physiological LysoPC Levels Are Conducive to Sexual Commitment (A) LysoPC levels drop during bacterial and parasitic infection in humans. The effect of different infections on serum LysoPC concentration is shown (Drobnik et al., 2003, Lakshmanan et al., 2012, Lamour et al., 2015, Ollero et al., 2011, Orikiiriza et al., 2017). CF, cystic fibrosis. (B) LysoPC levels drop during rodent malaria infection. LysoPC species were quantified in mice infected with *P. berghei* (parasitemia of 10%–14%) and in healthy control mice. (C) LysoPC levels in *P. berghei*-infected mice correlate negatively with parasite burden. Shown is the correlation between LysoPC concentration (different species are indicated) and parasitemia in peripheral blood of five mice. Correlation coefficients are indicated. Concentrations of LysoPC species are normalized to maximum concentration found across all mice (see Figure S5 for absolute quantities). Standard errors of the mean are shown. (D) Elevated BSA levels increase the IC50 of LysoPC for sexual commitment. Higher LysoPC concentrations are required to prevent parasite sexual commitment in presence of 3.9% BSA compared to 0.39% BSA. n *=* 3. Standard errors are shown. (E) LysoPC levels are lower in bone marrow compared to serum. Shown are LysoPC levels in serum and cell-free bone marrow extracts from healthy mice. n = 5. Standard errors of the means are shown. (F) Sexual differentiation rates of Pf2004/164tdTom parasites cultured in erythrocytes of different maturity. Parasites were allowed to invade into reticulocyte-enriched erythrocyte populations, and sexual differentiation was monitored for inducing (−SerM) and for non-inducing (−SerM/LysoPC and +SerM) conditions. Each data point represents a biological replicate (mean of technical triplicates). Sexual differentiation rates were normalized to a reticulocyte-negative control population cultured under inducing −SerM conditions. See also Figure S5.

**Figure S5:**
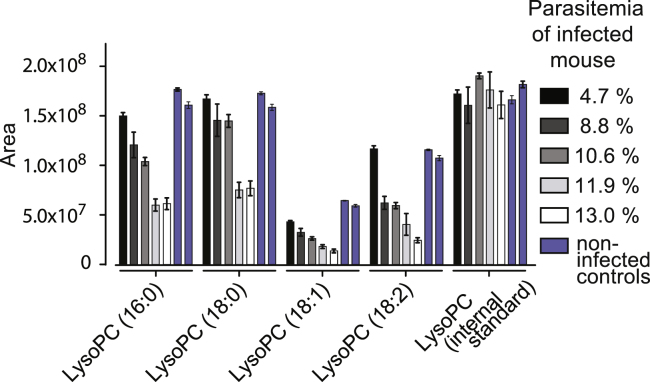
Related to Figure 6 LysoPC drops in response to parasite infection. Serum LysoPC levels were quantified in 5 mice infected with *P. berghei* and in 2 non-infected controls. LysoPC concentrations decrease in a parasitemia-dependent manner. Bars quantify area under the curve. Shown are standard errors of the means from 3 technical replicates.

LysoPC levels in human blood plasma are in the range of 20–100 μM, depending on the lipid species (Psychogios et al., 2011). Interestingly, these lipids are chemotactic factors of the innate immune system, and their plasma levels are significantly reduced in response to various severe infection conditions (Drobnik et al., 2003, Ollero et al., 2011) (Figure 6A). Two recent studies have demonstrated that various LysoPC species drop dramatically during malaria infection, coinciding with the pyrogenic peak (Lakshmanan et al., 2012, Orikiiriza et al., 2017) (Figure 6A). For example, the major LysoPC species in human serum, LysoPC (16:0) drops from 106.6 μM to 26.4 μM. Similar observations were made during infection with African trypanosomes (Lamour et al., 2015) that also utilize plasma-derived LysoPC for PC biosynthesis (Bowes et al., 1993). Given that rodent malaria parasites require LysoPC for normal growth (Figures 5C and 5D), we determined the levels of this lipid in *P. berghei*-infected mice. Metabolomic analysis of serum samples revealed that certain LysoPC species dropped by up to 76% compared to uninfected controls (Figures 6B and S5). In addition, we observed a negative correlation between LysoPC levels and parasite density in infected mice (Figure 6C), demonstrating that *Plasmodium* infection can alter systemic LysoPC levels via parasite metabolism, host inflammation responses, or likely a combination of the two.

Serum albumin is known to bind LysoPC, thereby regulating availability and biological activity of this phospholipid under physiological conditions (Kim et al., 2007). In line with this finding, we observed that increasing bovine serum albumin (BSA) levels from 0.39% (standard *P. falciparum in vitro* culture levels) to 3.9% (physiological levels) significantly increased the LysoPC (16:0) concentration required for preventing parasite sexual commitment (from 1.73 μM to 21.5 μM [95% CI 17.3–27.2 μM, Figure 6D]). Notably, this activity window is within the physiological range of LysoPC concentrations observed during malaria infection in humans and mice (Figures 6A and 6B). However, *in vitro* experiments can only provide an approximation of active LysoPC levels in the host, where lipoproteins and metabolic turnover likely reduces bioavailable LysoPC. Depending on the exact *in vivo* environment, parasites may therefore already induce sexual commitment prior to the extensive depletion of this lipid in serum.

Moreover, sequestration in both the intra- and extravascular spaces exposes parasites to microenvironments that show varying LysoPC levels. For instance, we found concentrations of LysoPC to be drastically reduced in bone marrow fluids compared to serum of healthy mice (Figure 6E), possibly because the majority of the LysoPC pool in plasma is bound to albumin (Kim et al., 2007), and this complex cannot easily cross the vascular barrier.

Altogether, these results demonstrate that *P. falciparum* parasites control sexual differentiation in response to LysoPC levels that naturally occur in the human host.

## Discussion

During their life cycle, malaria parasites are exposed to various host environments in the intermediate vertebrate and definitive arthropod host. They have evolved sophisticated mechanisms to sense external cues and adapt to changing environments. For example, parasite transmission to the arthropod host coincides with a drop in temperature and exposure to mosquito factors, triggering a final differentiation step into male and female gametes (Billker et al., 1998). The preceding switch from the asexually replicating blood stage to a gametocyte requires balancing the trade-off between maximizing within-host survival and between-host transmission under varying host conditions. This concept of reproductive restraint is the basis for the hypothesis that the switch from asexual to sexual parasite stages is environmentally sensitive (Pollitt et al., 2011). A number of physiological cues, as well as antimalarial drugs, have been implicated in affecting this switch, i.e. the rate of sexual differentiation. Observed effects were generally subtle and possibly the result of a general stress response and/or selective killing of asexual or sexual parasite stages (Buchholz et al., 2011). In contrast, CM results in a strong and reproducible increase in sexual conversion rates (Brancucci et al., 2015). We have previously demonstrated that part of this activity can be attributed to the presence of parasite-derived vesicles in CM (Mantel et al., 2013). Here, we used an optimized sexual conversion assay (Brancucci et al., 2015) to conclusively show that the major activity of CM derives from the depletion of the serum lipid LysoPC. Our data demonstrate that malaria parasites utilize LysoPC as a major substrate for phospholipid metabolism. In *P. falciparum*, limited LysoPC availability acts as an environmental sensor, reducing the number of daughter cells produced by blood-stage parasites and stimulating differentiation towards the transmissible gametocyte stage.

Comparative transcriptional profiling of *P. falciparum* parasites cultured in presence or absence of LysoPC revealed a cascade of pathways feeding into *ap2-g* activation, defining the earliest signature of parasite sexual commitment. For example, we observed that chromatin-modifying enzymes are induced in conditions that promote sexual differentiation. While these factors may be involved in epigenetic reprogramming, ultimately resulting in *ap2-g* activation, the identified cell-cycle regulators may prepare the cell for the G0-like state initiated during gametocyte development (Sinden et al., 1996). A series of merozoite antigens were also induced under LysoPC-limiting conditions, suggesting that sexually and asexually committed cells show diverging host-cell tropism. Single-cell transcriptomics will be required to de-convolve these transcriptional profiles and define the true signature of sexual versus asexual commitment, as well as to separate them from unrelated responses to nutrient depletion. Interestingly, several *P. falciparum* genes induced in absence of LysoPC are absent in the rodent malaria lineage, which may explain non-responsiveness of *P. berghei* to LysoPC-derived differentiation signals. One gene encodes an ApiAP2 transcription factor (*PF3D7_1222400*) located close to the *ap2-g* locus. Both transcription factors harbor frequent nonsense mutations in culture-adapted *P. falciparum* strains (Claessens et al., 2017, Kafsack et al., 2014), suggesting that their loss confers a fitness advantage under *in vitro* conditions, as has also been shown for the essential gametocytogenesis factor *gdv1* (Eksi et al., 2012). Likewise, *in vitro* conditions may also select for mutations that interfere with LysoPC sensing, especially when parasites are cultured under serum-free conditions such as those of Albumax-complemented medium (Srivastava et al., 2007).

We demonstrate that malaria parasites use LysoPC as a major source of choline and fatty acids for PC biosynthesis and that corresponding metabolic activity in the Kennedy pathway is directly linked to *P. falciparum* transmission-stage formation. Several scenarios may cause altered host LysoPC levels during infection, thereby triggering metabolic restriction and different degrees of sexual commitment. We first considered the possibility that different host cell types and tissue compartments contain different amounts of LysoPC. Consistent with a series of reports demonstrating increased gametocyte formation at higher reticulocyte densities (Peatey et al., 2013, Trager et al., 1999), we hypothesized that the metabolic needs of reticulocytes may restrict LysoPC availability to the parasite and hence trigger sexual differentiation. We tested this hypothesis by culturing *P. falciparum* in reticulocyte-enriched blood; however, we did not observe any difference in sexual commitment rates (Figure 6F). Our results rule out a direct effect of reticulocytes on gametocyte production and suggest that the observed correlation between high reticulocyte counts and/or anemia with increased gametocyte formation in malaria patients (e.g., Price et al., 1999) must arise from indirect host changes rather than from a direct effect of reticulocyte infection. Alternatively, commitment could be promoted in specific tissue compartments due to limited LysoPC availability. First, efficient LysoPC uptake and turnover by the parasite could generate a LysoPC sink contributing to increased local sexual commitment at sites of vascular sequestration. In support of this hypothesis, both symptomatic and asymptomatic *P. falciparum* infections produce detectable levels of *ap2-g*, but not immature gametocyte transcripts, in patient blood (Farid et al., 2017, Pelle et al., 2015). Second, we recently observed accumulation of immature *P. falciparum* gametocytes in the extravascular environment of the bone marrow (Aguilar et al., 2014, Joice et al., 2014), suggesting that early gametocytes form either in the vasculature before homing to bone marrow or directly in the bone marrow niche (Nilsson et al., 2015) or both. Indeed, we found that LysoPC levels are significantly reduced in bone marrow fluids compared to serum (Figure 6E). Therefore, decreased LysoPC concentration in bone marrow may trigger gametocytogenesis in a tissue-specific manner. Together, our data provide supportive evidence for mutually compatible scenarios where physiological variation of LysoPC levels regulates sexual differentiation during *P. falciparum* infection.

Not only do LysoPC levels differ spatially within a host, but systemic LysoPCs are also reduced in response to various severe infection conditions (Drobnik et al., 2003, Ollero et al., 2011), including human and rodent malaria and African trypanosomiasis (Lakshmanan et al., 2012, Lamour et al., 2015, Orikiiriza et al., 2017). Both *Plasmodium* (this study) and *Trypanosoma brucei* (Bowes et al., 1993) utilize plasma-derived LysoPC for PC biosynthesis. It is therefore possible that the synergistic effect of host immune response and parasite metabolism limits availability of systemic LysoPC, which increases gametocyte formation and malaria transmission, in particular during severe disease. Indeed, LysoPC levels decrease dramatically during the course of a fulminant mouse infection with *P. berghei*, and this decrease correlates with higher parasitemia (see also Figures 6B and 6C). A similar reduction of LysoPC levels was observed in CM of *P. falciparum in vitro* cultures compared to +SerM, demonstrating high LysoPC turnover by parasites independently of host-mediated processes. Altogether, these findings provide the basis for testable models describing the parasite’s capability to vary growth and transmission in response to altering host conditions.

Finally, the observation that LysoPC restriction results in reduced progeny numbers emphasizes the importance of phospholipid metabolism for parasite growth and survival. Indeed, several key enzymes of the parasite Kennedy pathway have been recognized as attractive antimalarial drug targets, including the previously discussed PfPMT (Bobenchik et al., 2010, Pessi et al., 2004) and PfEK, as well as PfCK (Serrán-Aguilera et al., 2016) and PfCCT (CTP: phosphocholine cytidylyltransferase) (González-Bulnes et al., 2011). While our data revealed that limiting LysoPC levels induce PfPMT expression specifically in sexually committed cells, inhibition of this enzyme, either by genetic disruption or using specific inhibitors, also blocks gametocyte maturation and transmission to mosquitos (Bobenchik et al., 2013). In addition, analogs of the substrate choline show high efficacy against *P. falciparum* blood-stage parasites, and several are currently tested in clinical trials (Peyrottes et al., 2012). Our findings reinforce the observation that parasite phospholipid biosynthesis represents a promising multi-stage target for antimalarial drug development (Ben Mamoun et al., 2010).

In summary, dropping levels of host LysoPC can trigger metabolic and transcriptional changes that initiate sexual commitment and subsequent gametocytogenesis in *P. falciparum*. This finding represents the first described host factor linking environmental sensing to metabolic adaptation and cell-fate determination in response to changing host conditions in a eukaryotic pathogen. The fundamental role of LysoPC in *P. falciparum* survival, differentiation, and transmission offers unique opportunities for novel interventions in the fight to eradicate malaria.

## STAR★Methods

### Key Resources Table

**Table:** 

REAGENT or RESOURCE	SOURCE	IDENTIFIER
**Antibodies**		

Anti-PMT antibody	Bobenchik et al., 2013	N/A
CD71-APC, human (clone: AC102)	Miltenyi Biotec	Cat#130-091-727; RRID: AB_615103

**Chemicals, Peptides, and Recombinant Proteins**		

Phosphatidylcholine (PC) 16:0-16:0	Avanti Polar Lipids	Cat#850355
PC 16:0-18:1	Avanti Polar Lipids	Cat#850457
LysoPC 16:0	Avanti Polar Lipids	Cat#855675
LysoPC 18:0	Avanti Polar Lipids	Cat#855775
LysoPC 18:1	Avanti Polar Lipids	Cat#845875
Phosphatidic acid (PA) 16:0-18:1	Avanti Polar Lipids	Cat#840857
PA 18:1-18:1	Avanti Polar Lipids	Cat#840875
PE 16:0-16:0	Avanti Polar Lipids	Cat#850705
Diacylglycerol (DG) 16:0-16:0	Avanti Polar Lipids	Cat#800816
DG 16:0-18:1	Avanti Polar Lipids	Cat#800815
Edelfosine	Avanti Polar Lipids	Cat#999995
Miltefosine	Avanti Polar Lipids	Cat#850337
LysoPAF	Avanti Polar Lipids	Cat#878119
TopFluor LysoPC	Avanti Polar Lipids	Cat#810284
TopFluor PC	Avanti Polar Lipids	Cat#810281
NBD-PA	Avanti Polar Lipids	Cat#810174
SPLASHTM Lipidomix standard mix	Avanti Polar Lipids	Cat#330707
Palmitic acid-1,2-13C	Cambridge Isotope Laboratories	Cat#CLM-214
ER tracker red (Bodipy TR glibenclamide)	Invitrogen	Cat#E34250
BR23	Serrán-Aguilera et al., 2016	N/A
Phosphocholine chloride calcium salt	Sigma Aldrich	Cat#P0378
choline chloride-(trimethyl-d9)	Sigma Aldrich	Cat#492051
S-(–)-glycidol	Sigma Aldrich	Cat#474789
N,N-Diisopropylethylamine	Sigma Aldrich	Cat#D125806
Phosphorus(V) oxychloride	Sigma Aldrich	Cat#201170
Pyridine	Sigma Aldrich	Cat#270970
Concanavalin A	Sigma-Aldrich	Cat#C5275
Bovine Serum Albumin (essentially fatty acid free)	Sigma-Aldrich	Cat#A6003
Oleic acid	Sigma-Aldrich	Cat#O1008
Palmitic acid	Sigma-Aldrich	Cat#P0500
Thiazole orange	Sigma	Cat#390062

**Critical Commercial Assays**		

High Sensitive D1000 Reagents	Agilent Technologies	Cat#5067-5585
High Sensitive RNA Screen sample buffer	Agilent Technologies	Cat#5067-5580
Agencourt AMPure XP beads	Beckman Coulter	Cat#A63881
MiSeq Reagent Kit v2 (300 cycle)	Illumina	Cat#MS-102-2002
TruSeq Stranded mRNA library Prep Kit	Illumina	Cat#RS-122-2101
PhiX Control Kit v3	Illumina	Cat#FC-110-3001
Library Quantification Kit, Complete kit	KAPA BIOSYSTEMS	Cat#KK4835
SuperScript II Reverse Transcriptase	Life Technologies	Cat#18064014
DNase I	Life Technologies	Cat#18068-015

**Deposited Data**		

Raw and analyzed RNA-seq data	This paper	GEO: GSE104114

**Experimental Models: Cell Lines**		

Parasite strain: *Plasmodium falciparum* Pf2004/164tdTom	Brancucci et al., 2015	N/A
Parasite strain: *Plasmodium falciparum* HB3/748tdTom	Brancucci et al., 2015	N/A
Parasite strain: *Plasmodium falciparum* NF54/AP2-G^GFP^	This paper	N/A
Parasite strain: *Plasmodium berghei* ANKA gametocyte reporter line	This paper and Sinha et al., 2014	G1137cl2
Parasite strain: *Plasmodium berghei* ANKA PbmCherryHsp70	Burda et al., 2015	PbANKA mCherryHsp70 (pL1694)
Mouse strain: C57BL/6JOlaHsd	Harlan UK Limited	050

**Experimental Models: Organisms/Strains**		

Mouse: Theiler’s Original (female)	Envigo	N/A

**Oligonucleotides**		

Oligonucleotides used in this study are provided in Table S1	This study	N/A

**Software and Algorithms**		

Agilent MassHunter Workstation	Agilent Technologies	N/A
Agilent MassHunter Qualitative Analysis	Agilent Technologies	N/A
FlowJo 10	FLOWJO, LLC	N/A
Prism 6	Graphpad	N/A
TopHat	Kim et al., 2013	http://ccb.jhu.edu/software/tophat/index.shtml RRID: SCR_013035
Fiji (fluorescence intensity measurement functions)	Open Source; Schindelin et al., 2012	http://imagej.net/Fiji/Downloads RRID: SCR_002285
Cufflinks	Trapnell et al., 2010	http://cole-trapnell-lab.github.io/cufflinks/ RRID: SCR_014597
R	The R project	https://www.r-project.org/ RRID: SCR_001905

**Other**		

MACS LD columns	Miltenyi Biotec	Cat#130-042-901

### Contact for Reagent and Resource Sharing

Further information and requests for reagents should be directed to and will be fulfilled by the Lead Contact, Matthias Marti (matthias.marti@glasgow.ac.uk).

### Experimental Model and Subject details

#### Mouse Model

Mice used in this study (Theiler’s original naive mice, TO; age 6-8 weeks; weight 25-30g) were maintained according to Home Office license (60/4443) regulations. *P. berghei* infections for merozoite counts and sexual differentiation assays were performed on female Theiler’s original mice (TO; age 6-8 weeks; weight 25-30 g). *P. berghei* infections for serum extraction and metabolic analysis of correlations of LysoPC levels and peripheral parasitemias were performed on female C57BL/6 mice (6-8 weeks). Naïve mice received an initial inoculum of 10^6^ parasites of the *P. berghei* ANKA line PbmCherry_Hsp70_ (Burda et al., 2015), and were exsanguinated by cardiac puncture at day 6 post infection to isolate serum.

#### Parasite Cultures

*P. falciparum: P. falciparum* cell culture and synchronization was performed as described (Lambros and Vanderberg, 1979, Trager and Jensen, 1976). Cultures were kept at 37°C in RPMI-1640 medium supplemented with 25 mM HEPES, 100 μM hypoxanthine (all from Sigma-Aldrich), with 10% human serum (The Interstate Companies), 24 mM sodium bicarbonate (Sigma-Aldrich) and gassed with 5% CO_2_/1% O_2_ and 94% N_2_ mixture, unless otherwise stated. Pf2004/164tdTom and HB3/748tdTom parasites were grown in the presence of 4 nM WR99210 (Jacobus Pharmaceuticals) to select for stable episomes. The Pf2004 strain was transfected with reporter plasmid 164tdTom to generate Pf2004/164tdTom. Consequently, this cell line expresses the fluorescent protein tdTomato under control of the gametocyte-specific promoter of PF3D7_1016900 (Brancucci et al., 2015, Buchholz et al., 2011). Similarly, the HB3 strain used in this study (HB3/748tdTom) expresses the tdTomato reporter under control of gametocyte-specific promoter of PF3D7_1477700 (Buchholz et al., 2011). Experiments were carried out using a clonal Pf2004/164tdTom population, obtained by limiting dilution. Parasites of this cell line predominantly form asexual progeny (>99%) when cultured in presence of 10% serum (Brancucci et al., 2015). Sex ratios were not determined in this study. Serum-free medium (−SerM) was generated by complementing RPMI-1640 (prepared as described above, w/o serum) with 0.39% fatty acid-free BSA and oleic and palmitic acid (30 μM each; added from 30 mM ethanol-solved stocks; all from Sigma-Aldrich). LysoPC (Avanti Polar Lipids) was added to this medium to a final concentration of 20 μM (or the indicated concentration) to generate −SerM/LysoPC medium.

*P. berghei: P. berghei* parasites were cultured *ex vivo* at 37°C in RPMI1640 supplemented with 25 mM HEPES, 100 μM hypoxanthine, 20% FCS, 10 mM sodium bicarbonate, 100 U/mL penicillin and 100 μg/mL streptomycin (all from Sigma-Aldrich) and gassed with 5 CO_2_/5% O_2_ and 90% N_2_ mixture. For mouse serum extraction and LysoPC quantification experiments, parasitemia was calculated prior to exsanguination. To this end, a drop of blood was obtained by tail vein puncture and the proportion of fluorescent (infected) red blood cells was quantified by flow cytometry.

### Method Details

#### General analytical methods

HPLC was performed with an Agilent 1200 series HPLC system (Agilent Technologies) equipped with a photo-diode array detector; all solvents were HPLC grade. Low-resolution Mass Spectrometry data were obtained using the above system and a 6130 quadrupole mass spectrometer; all solvents were LC-MS grade. High-resolution Mass Spectrometry data were obtained using an Agilent 1290 LC system equipped with an Agilent 6530 QTOF mass spectrometer in full scanning positive or negative ion mode in a range of 100-1700 m/z. Instrument conditions were as follows: Gas temperature at 325°C, gas flow at 10 L/min, Nebulizer at 40 psi, Capillary (positive) at 3500 V, Fragmentor at 120 V, and Skimmer at 65 V. Synthetic standards were used to establish retention times and characteristic ionization of lipid classes (SPLASH Lipidomix, Avanti Polar Lipids; choline chloride, Sigma-Aldrich; and phosphocholine chloride calcium salt, Sigma-Aldrich).

#### Serum fractionation

Human serum (45 mL) was split evenly into 4 x 50 mL polypropylene centrifuge tubes. Each tube was extracted with 25 mL of chloroform/methanol (2:1). After collecting and pooling the bottom layers, the top layers were re-extracted with 20 mL chloroform. The bottom layers were combined with the bottom layers from the first extraction, filtered, and vacuum concentrated. This lipid extract was dissolved in 0.5 mL of chloroform and split into two aliquots. Each of these 250 μL aliquots was passed through an aminopropyl solid phase extraction column (Supelco DSC-NH_2_, 1 g) that had been conditioned with 6 mL of hexanes. After loading the lipid extract, the columns were eluted with 5 mL each of (1) hexanes, (2) hexanes/dichloromethane/diethyl ether (89:10:1), (3) hexanes/ethyl acetate (85:15), (4) chloroform/methanol (2:1), (5) methanol, (6) methanol with 1% ammonium hydroxide, (7) methanol with 1% acetic acid, (8) water with 10% ammonium hydroxide. The common fractions from the two columns were combined and concentrated *in vacuo*.

Activity of each fraction was tested by dissolving the fractions in 4 mL of mixtures of chloroform and methanol. These solutions were added in 2.2 μL aliquots to a glass bottom 96-well plate, the solvent was evaporated, and 220 μL of parasite culture in CM (80% parasite-conditioned medium; as described (Brancucci et al., 2015)) was added to the residue. Fraction (4) was the most active. It contained many lipid species, so it was further fractionated by HPLC. Half of the fraction (16 mg) was fractionated by HPLC using a Phenomenex Luna C18 column with a flow rate 0.7 mL/min. Solvent A was water + 0.1% formic acid and solvent B was acetonitrile + 0.1% formic acid. After a 5-minute equilibration of 80% of solvent B, a 20 minute gradient was run up to 100% of solvent B. Forty fractions of 0.5 to 1 minute were collected. Identical fractions from 15 HPLC runs were pooled, concentrated *in vacuo*, and re-dissolved in 200 μL methanol. Fractions were tested for blocking sexual differentiation as described below. Activity was found localized to two sets of fractions, (1) fractions 13-16, and (2) fractions 23-24. These active fractions were investigated by high-resolution LC-MS in the positive mode using a Phenomenex Kinetex EVO C18 column. Solvent A was water and solvent B was acetonitrile. A 12-minute gradient of 80% of solvent B up to 100% of solvent B was utilized. The main components in fractions 13-16 and 23-24 were LysoPCs (in fractions 13-16 LysoPC(16:0); in fractions 23-24 LysoPC(18:0), see Figures 2B and S1B). To confirm the activity of LysoPCs, we purchased LysoPC(16:0), LysoPC(18:0) and LysoPC(18:1) from Avanti Polar Lipids and tested them in the previously described gametocytogenesis assay. All three species inhibited gametocytogenesis at 20 μM.

#### Analysis of LysoPC levels in mice

Serum aliquots from each sample were split into three 20 μL portions in glass vials. To each portion was added 4 μL of a 500 μM solution of 1-*O*-palmitoyl-sn-glycero-3-phosphocholine(d_9_) (compound 4, 2000 pmol total*)* in methanol as an internal standard. The samples were randomized and extracted via a modified Bligh and Dyer method (Bligh and Dyer, 1959). To each sample was added 0.5 mL of 2 M NaCl and 1.9 mL of 1:2 chloroform/methanol. After mixing the samples by vortex and incubation at room temperature for 15 minutes, 0.6 mL of water and 0.6 mL of chloroform were added. The layers were separated by centrifugation, and the lower layer was collected. The upper layer was re-extracted with another 0.8 mL of chloroform, and the lower layer was collected and combined with the first. The samples were dried in a speedvac concentrator, and redissolved in 75 μL of 2:1 isopropanol/methanol. High-resolution LC-MS was performed in both positive mode and negative mode using a Phenomenex Luna C5 column. Solvent A was 95% water, 5% methanol, 0.1% formic acid, and 5 mM ammonium formate; solvent B was 60% isopropanol, 35% methanol, 5% water, 0.1% formic acid, and 5 mM ammonium formate. After injection of 5 μL, solvent A was pumped at 0.1 mL/min for five minutes, after which the flow rate was increased to 0.4 mL/min, and a 40-minute linear gradient was run from 20% solvent B to 100% solvent B. The LysoPC peaks were quantified by integration of [M+H]+ peaks in Agilent MassHunter software and plotted against parasitemia.

#### Comparison of LysoPC in mouse serum and bone marrow

Analysis was performed essentially as described above with the following exceptions. One 3 μL aliquot of serum from each of five mice was diluted to 0.2 mL and added to 0.3 mL of 2M NaCl (instead of 20 μL aliquots added to 0.5 mL of 2 M NaCl). Cell-free bone marrow dilutions were obtained by extracting bone marrow from the femur and tibia of both lower extremities of each mouse. The bone marrow was collected in 0.5 mL of PBS, and its weight recorded using a high sensitivity scale, prior to disruption using an insulin needle followed by centrifugation at 10,000 g for 5 minutes. The supernatant was recovered and weighed following centrifugation to determine the percentage of the collected material. A 0.2 mL aliquot of the diluted cell-free bone marrow from each of five mice was added to 0.3 mL of 2 M NaCl. To all 10 samples (5 serum, 5 bone marrow) 4 μL of a 25 μM solution of 1-*O*-palmitoyl-sn-glycero-3-phosphocholine(d_9_) (compound 4, 100 pmol total*)* were added in methanol as an internal standard. Extractions and LC-MS analyses were performed as described above. To calculate the concentration of LysoPCs in undiluted bone marrow, each value was multiplied by the dilution factor (200x).

#### Synthesis of labeled LysoPC(16:0) species

##### Overview:

Two heavy-atom labeled LysoPC(16:0) analogs (d_9_-choline and ^13^C_2_-palmitate) were synthesized following the protocol published by Lindberg *et al.* (Lindberg et al., 2002):

##### Labeled LysoPC(16:0) analogs:

**Figure:**
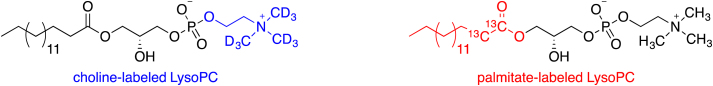

##### General reaction scheme:

**Figure:**
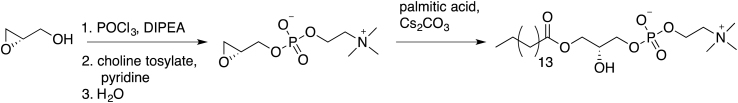

Generally, reactions were carried out in flame-dried glassware under Argon. Anhydrous solvents were purchased from Sigma-Aldrich. ^13^C_2_-labeled palmitic acid was purchased from Cambridge Isotope Laboratories, and d_9_-labeled choline chloride was purchased from Sigma-Aldrich. All other reagents were also purchased from Sigma-Aldrich. Choline tosylate was produced from choline chloride by passing through ion exchange resin (Amberlite IRN78, hydroxyl form) followed by acidification with *p*-toluenesulfonic acid and crystallization from acetone.

##### Synthesis of (R)-glycidyl phosphocholine (1):

**Figure:**
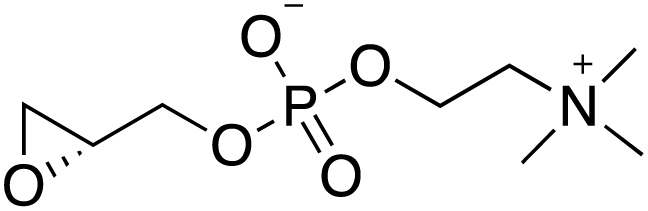

S-glycidol (0.60 mL, 0.67 g, 9.0 mmol) and *N,N*-Diisopropylethylamine (DIPEA, 6.5 mL, 4.8 g, 9.5 mmol) were added to 20 mL anhydrous CHCl_3_. Phosphorus(V) oxychloride (POCl_3_, 0.85 mL, 1.4 g, 9.1 mmol) was added dropwise to the solution stirring in an ice-water bath. After stirring at 0°C for 2 hours, unlabeled choline tosylate (3.0 g, 10.6 mmol) was added, followed by pyridine (2.0 mL, 2.0 g, 25 mmol). After pyridine addition, the reaction turned magenta. The flask was removed from the ice-water bath and allowed to stir at room temperature for 5 hours. The reaction was quenched with 1.0 mL water and allowed to stir at room temperature for 1 hour. The mixture was concentrated by rotary evaporation. Almost half of this crude product (6.0 g out of 13.5 g) was purified by silica gel chromatography, eluting with 70% ethanol in water (Rf ∼0.2). Fractions containing the product were combined, concentrated by rotary evaporation, and centrifuged to remove particles. The supernatant was concentrated to yield thick oil (R)-glycidyl phosphocholine (compound 1, 400 mg, 1.67 mmol, 42%).

^1^H NMR (400 MHz, Methanol-d4) δ 2.68 (dd, J = 5.0, 2.7 Hz, 1H), 2.81 (ddd, J = 5.0, 4.2, 1.0 Hz, 1H), 3.19 – 3.27 (s, 9H & m, 1H), 3.62 – 3.67 (m, 2H), 3.67 – 3.77 (m, 1H), 4.18 (dddd, J = 12.0, 6.9, 2.7, 0.7 Hz, 1H), 4.23 – 4.34 (m, 2H); ^13^C NMR (100 MHz, Methanol-d4) δ 44.99, 51.96 (d, J = 8.3 Hz), 54.66, 54.70, 54.74, 60.42 (d, J = 5.2 Hz), 67.37 – 67.59 (m), 67.72 (d, J = 5.5 Hz).

MS (single quadrupole): calculated for C_8_H_18_NO_5_P: 240.1 [M+H]+; found: 240.2 [M+H]+.

##### Synthesis of (R)-glycidyl phosphocholine(d_9_) (2):

**Figure:**
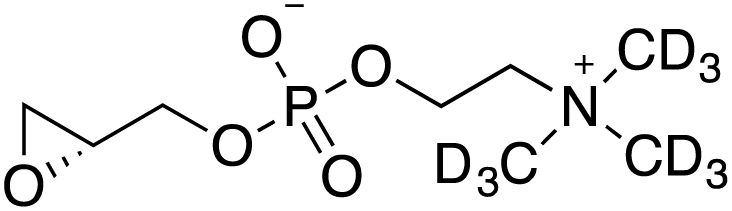

S-glycidol (0.20 mL, 0.22 g, 3.1 mmol) and *N,N*-Diisopropylethylamine (DIPEA, 2.17 mL, 1.6 g, 12.5 mmol) were added to 7 mL anhydrous CHCl_3_. Phosphorus(V) oxychloride (POCl_3_, 0.28 mL, 0.46 g, 3.0 mmol) was added dropwise to the solution stirring in an ice-water bath. After stirring at 0°C for 2 hours, d_9_-labeled choline tosylate (1.0 g, 3.5 mmol) was added, followed by pyridine (0.67 mL, 0.66 g, 8.3 mmol). After pyridine addition, the reaction turned magenta. The flask was removed from the ice-water bath and allowed to stir at room temperature for 3 hours. The reaction was quenched with 0.3 mL water and allowed to stir at room temperature for 1 hour. The mixture was concentrated by rotary evaporation and purified by silica gel chromatography, eluting with 70% ethanol in water. Fractions containing the product were combined, concentrated by rotary evaporation, and centrifuged to remove particles. The supernatant was concentrated to yield thick oil (R)-glycidyl phosphocholine(d_9_) (compound 2, 323 mg, 1.30 mmol, 42%).

^1^H NMR (400 MHz, Methanol-d4) δ 2.68 (dd, J = 5.0, 2.7 Hz, 1H), 2.81 (t, J = 4.6 Hz, 1H), 3.19 – 3.27 (m, 1H), 3.61 – 3.67 (m, 2H), 3.67 – 3.76 (m, 1H), 4.19 (ddd, J = 12.0, 6.9, 2.6 Hz, 1H), 4.24 – 4.38 (m, 2H); ^13^C NMR (100 MHz, Methanol-d4) δ 45.02, 51.99 (d, J = 8.1 Hz), 53.48 (weak), 53.70 (weak), 53.93 (weak), 60.40 (d, J = 5.1 Hz), 67.33 – 67.01 (m), 67.72 (d, J = 5.4 Hz).

MS (single quadrupole): calculated for C_8_H_9_D_9_NO_5_P: 249.2 [M+H]+; found: 249.1 [M+H]+.

##### Synthesis of 1-O-palmitoyl(1,2-^13^C_2_)-sn-glycero-3-phosphocholine (3):

**Figure:**
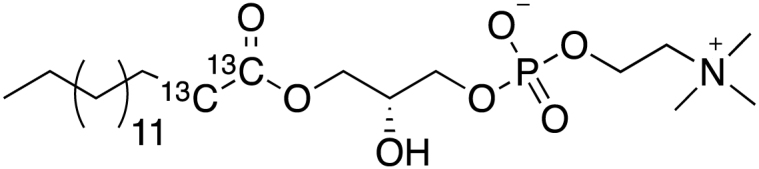

Palmitic acid-1,2-^13^C_2_ (206 mg, 0.80 mmol), cesium carbonate (Cs_2_CO_3_, 265 mg, 0.81 mmol), and the unlabeled (*R*)-glycidyl phosphocholine (1) (200 mg, 0.84 mmol) were dissolved in 10 mL dimethylformamide and stirred at 80°C for 6.5 hours. The reaction mixture was cooled on ice and added to 40 mL of 20% methanol in water, which was loaded onto a C18 Seppak cartridge (Waters WAT043345, 35 cc, 10 g) that had been activated with methanol and equilibrated with 20% methanol in water. The cartridge was eluted with 20 mL each of 20%, 50%, and 80% methanol in water, and finally with 30 mL 100% methanol. The product 1-O-palmitoyl(1,2-^13^C_2_)-sn-glycero-3-phosphocholine was present in the 100% methanol elution, which was further purified by reverse phase HPLC using a phenyl-hexyl resin (Phenomenex 00G-4257-N0, Luna 5 μm, 100 Å, 250 x 10 mm). Solvent A was water, and solvent B was acetonitrile. After a 5 minute equilibration at 50% B, a 15 minute gradient from 50% B to 100% B eluted pure product 1-O-palmitoyl(1,2-^13^C_2_)-sn-glycero-3-phosphocholine (3), which was concentrated to yield a white powder (48 mg, 0.096 mmol, 12%)

^1^H NMR (400 MHz, Methanol-d4) δ 0.90 (t, 2H, *J* = 7.0 Hz), 1.29 (m, 24H), 1.62 (m, 2H), 2.35 (dq, 2H, *J* = 128, 7.5 Hz), 3.23 (s, 9H), 3.64 (m, 2H), 3.85–3.93 (m, 2H), 3.93–4.00 (m, 1H), 4.11 (ddd, 1H, *J* = 11.4, 6.1, 3.1 Hz), 4.18 (ddd, 1H, *J* = 11.4, 4.4, 2.7 Hz), 4.29 (m, 2H); ^13^C NMR (100 MHz, Methanol-d4) δ 14.4, 23.7, 26.0 (dd, *J* = 34.5, 1.5 Hz), 30.2 (d, *J* = 3.8 Hz), 30.4 (d, *J* = 4.1 Hz), 30.5, 30.6, 30.7–30.8 (multiple peaks), 33.1, 34.9 (d, *J* = 57.5 Hz), 54.64, 54.68, 54.72, 60.4 (d, *J* = 5.0 Hz), 66.2 (d, *J* = 2.5 Hz), 67.5 (m), 67.8 (d, *J* = 5.8 Hz), 69.9 (dd, *J* = 7.6, 2.2 Hz),175.3 (d, *J* = 57.5 Hz).

MS (ESI-TOF): calculated for C_22_^13^C_2_H_50_NO_7_P: 498.3465 [M+H]+; found: 498.3460 [M+H]+.

##### Synthesis of 1-O-palmitoyl-sn-glycero-3-phosphocholine(d_9_) (4):

**Figure:**
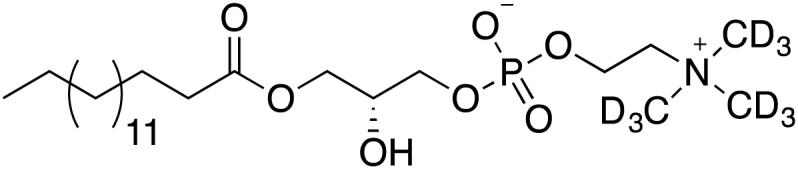

Unlabeled palmitic acid (215 mg, 0.84 mmol), cesium carbonate (Cs_2_CO_3_, 270 mg, 0.82 mmol), and the (*R*)-glycidyl phosphocholine (1) (125 mg, 0.50 mmol) were dissolved in 10 mL dimethylformamide and stirred at 80°C for 6 hours. The reaction mixture was worked up and purified as described above to yield pure product 1-O-palmitoyl-sn-glycero-3-phosphocholine(d_9_) (compound 4) as a white powder (53 mg, 0.11 mmol, 21%)

^1^H NMR (400 MHz, Methanol-d4) δ 0.90 (t, 2H, *J* = 7.0 Hz), 1.29 (m, 24H), 1.62 (quint, 2H, *J* = 7.3 Hz), 2.35 (t, 2H, *J* = 7.5 Hz), 3.63 (m, 2H), 3.85–3.93 (m, 2H), 3.93–4.00 (m, 1H), 4.11 (dd, 1H, *J* = 11.4, 6.1 Hz), 4.18 (dd, 1H, *J* = 11.4, 4.4 Hz), 4.29 (m, 2H); ^13^C NMR (100 MHz, Methanol-d4) δ 14.4, 23.7, 26.0, 30.2, 30.4, 30.5, 30.6, 30.7–30.8 (multiple peaks), 33.1, 34.9, 60.4 (d, *J* = 5.0 Hz), 66.2, 67.2 (m), 67.8 (d, *J* = 5.8 Hz), 69.9 (d, *J* = 7.6 Hz), 175.4.

MS (ESI-TOF): calculated for C_24_H_41_D_9_NO_7_P: 505.3963 [M+H]+; found: 505.3958 [M+H]+.

##### Synthesis of 1-O-Palmitoyl-2-methoxy-glycero-3-phosphocholine (5):

**Figure:**
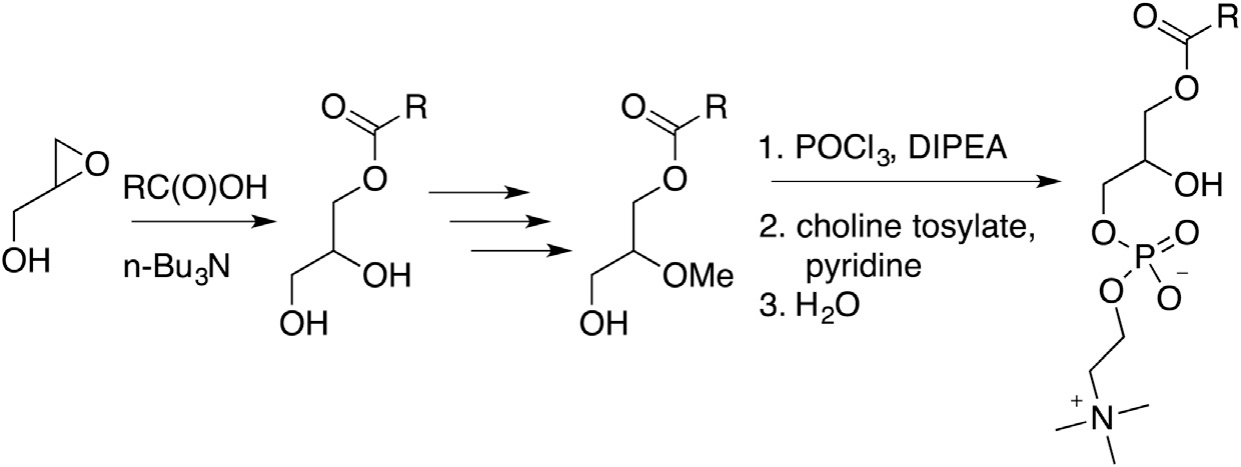

*OMe*-LysoPC analog (compound 5) was synthesized by modification of the procedure described by Lindberg *et al.* (Lindberg et al., 2002). The starting material 1-*O*-palmitoyl-2-methoxyglycerol was prepared from racemic glycidol as described (Rytczak et al., 2013). Phosphorus oxychloride (0.155 mL, 1.66 mmol) was added dropwise at 0°C to a solution of 1-*O*-palmitoyl-2-*methoxy*-glycerol 280 mg, 0.83 mmol) and ethyl *N,N*-diisopropylamine (0.29 mL, 1.66 mmol) in dichloromethane (10 mL) with constant stirring. After 2 hours, pyridine (0.27 mL, 3.32 mmol) and choline tosylate (457 mg, 1.66 mmol) were added, and the mixture was allowed to attain room temperature. The reaction mixture was stirred overnight at room temperature. Next, 0.2 mL water were added, and stirred for an additional 1.5 hours. After evaporation, the crude product was purified by silica gel flash column chromatography with a chloroform-methanol gradient (from 30 to 100% MeOH, v/v) followed by a methanol-water gradient (from 0 to 50% water, v/v) as eluent. The final *1-O-palmitoyl*-2-*OMe*-LPC (compound 5) was isolated after lyophilization. Yield 105.7 mg (25%). ^1^H NMR (250 MHz, Methanol-d4) *δ* 0.91 (t, 3H, *J* 6.9 Hz), 1.31 (br s, 24H), 1.60 (m, 2H), 2.38 (t, 2H, *J* 7.5 Hz), 3.29 (s, 9H), 3.50 (s, 3H), 3.66 (t, 2H, J 4.6 Hz), 3.99 (m, 2H), 4.1-4.34 (m, 3H); ^31^P NMR (Methanol-d4) *δ* 1.47. MALDI TOF MS: calculated MW for C_25_H_52_NO_7_P 509.65 Da; found: (M+H)+ *m/z* 510.5.

#### Metabolism of labeled LysoPC and choline

##### Incorporation experiments:

To test incorporation and metabolism of exogenously added LysoPC, *P. falciparum* parasites were grown for 12 hours (28±2 hpi to 40±2 hpi) in −SerM medium (100 mL, 5% hematocrit, 2% parasitemia) either in the absence or presence of 20 μM LysoPC(16:0) (Avanti Polar Lipids), 20 μM ^13^C_2_-palmitate-labeled-LysoPC(16:0) (synthesized above, compound 3), or 20 μM d_9_-choline-labeled-LysoPC(16:0) (synthesized above, compound 4). Cultures were pelleted by centrifugation for 5 min at 300 g. Erythrocytes were lysed in ice-cold 0.015% saponin (Sigma-Aldrich) in PBS for 10 min. After pelleting (5 min, 3000 g, 4°C), parasites were washed 5 times in 10 mL ice cold PBS. After the final wash, the parasite pellet was snap frozen in liquid nitrogen and stored at -80°C.

The same procedure was followed for characterizing incorporation and metabolism of exogenously added choline. Parasites were grown in −SerM medium either in the absence or presence of 1 mM choline chloride (Sigma-Aldrich) or 1 mM d_9_-choline chloride (Sigma-Aldrich).

##### Analysis of lipids:

Pellets were extracted following a previously described protocol (Gulati et al., 2015). Briefly, pellets were thawed, diluted with 600 μL water, split into 3 aliquots of 100 μL each in glass vials, and their order was randomized. To each 100 μL aliquot 1.33 mL of 3 M KCl and 0.5 mL methanol were added. Samples were vortexed and sonicated to homogeneity, and then extracted with 2 mL of isopropanol/hexanes (1:2). The top layer was collected and the bottom layer was re-extracted with 2 mL isopropanol/hexanes. The combined organic layers were dried *in vacuo*, resuspended in 500 μL hexanes, dried, resuspended in 300 μL methanol, dried, and resuspended in 200 μL chloroform for LC-MS analysis (5 μL injections). High-resolution LC-MS was performed in both positive mode and negative mode using a Phenomenex Luna C5 column. Solvent A was 95% water, 5% methanol, 0.1% formic acid, and 5 mM ammonium formate; solvent B was 60% isopropanol, 35% methanol, 5% water, 0.1% formic acid, and 5 mM ammonium formate. After injection, solvent A was pumped at 0.1 mL/min for five minutes, after which the flow rate was increased to 0.4 mL/min, and a 40-minute linear gradient was run from 20% solvent B to 100% solvent B. Peak areas of lysophosphatidylcholines, phosphatidylcholines, phosphatidylethanolamines, sphingomyelins, and ceramides were quantified by integration of [M+H]+ peaks in Agilent MassHunter software. Peak areas of fatty acids, diglycerides, and triglycerides were quantified by integration of [M+NH_4_]+ peaks, and monoglycerides were quantified by integration of [M+Na]+ peaks in Agilent MassHunter software. Peak areas of phosphatidic acids, phosphatidylserines, phosphatidylglycerols, and phosphatidylinositols were quantified by integration of [M–H]– peaks in Agilent MassHunter software.

##### Analysis of choline and phosphocholine:

Diluted pellets from the lipid analysis above were split into 3 aliquots of 75 μL each and their order was randomized. To each 75 μL aliquot in a 1.75 mL centrifuge tube was added 1.0 mL of ice-cold 80% methanol in water. These mixtures were kept on dry ice for 5 minutes, then vortexed one minute, kept on dry ice for 10 minutes, and vortexed again for one minute. The tubes were centrifuged at 4°C for 5 minutes at 18’000 g. The supernatant was collected, and the pellets were re-extracted with 1.0 mL of dry-ice-cooled 80% methanol in water. After pelleting again, the supernatants were combined and concentrated *in vacuo*. The residue was re-dissolved in 90% acetonitrile in water for LC-MS analysis (10 μL injections). High-resolution LC-MS was performed in positive mode using a Phenomenex Kinetex HILIC column. Solvent A was acetonitrile; solvent B was 5 mM ammonium formate in water. After injection, solvent A was pumped at 0.1 mL/min for five minutes, after which the flow rate was increased to 0.4 mL/min, and a 40-minute linear gradient was run from 20% solvent B to 100% solvent B. Peak areas of choline and phosphocholine were quantified by integration of [M+H]+ peaks in Agilent MassHunter software.

#### Quantification of sexual differentiation

Sexual differentiation was induced in tightly synchronized *P. falciparum* parasites (28±2 hpi) by incubating the cells for 22 hours in CM (220 μL per well of a 96-well plate; 0.3-0.5% parasitemia; 2.5% hematocrit) or in −SerM as described (Brancucci et al., 2015). If not stated otherwise, cell line Pf2004/164tdTom was used for all experiments. To determine the effect of culture perturbations on sexual commitment, serum fractions as well as nutrients or inhibitor compounds (solved in either RPMI, DMSO, chloroform, ethanol or methanol) were added to the bottom of empty wells (glass bottom dishes were used for chloroform-containing samples) and directly resuspended in parasite culture after allowing volatile solvents to evaporate. To determine sexual differentiation in reticulocyte-enriched blood, tightly synchronized parasites were magnet purified at 46±2 hpi using MACS CS columns in a SuperMACS (Miltenyi Biotec) before incubating pure schizont-infected erythrocytes (>99%) with the blood sample to be tested. These culture perturbations were then tested for effects on parasite sexual differentiation as described (Brancucci et al., 2015). In brief, following the 22 hour testing phase (see above), cells of each well were washed 3 times in 200 μL +SerM medium before being resuspended in 220 μL +SerM medium. Henceforth, medium was exchanged daily. Parasitemia and gametocytemia was quantified using flow cytometry at 20-30 hpi (MACS Quant, Analyzer 10) and 72-96 hpi (BD Fortessa), respectively. Cytometry data were analysed using FlowJo software and sexual differentiation rates were determined by dividing gametocytemia of each well with the corresponding parasitemia measurements. Assays were run in biological triplicates. Each biological replicate contained technical triplicates.

*P. berghei* sexual commitment assays were performed using a parasite line expressing an RFP reporter under the gametocyte-specific gene *PBANKA_1018700* (Sinha et al., 2014) and GFP under the constitutive *PBANKA_0905600* promoter, in the 507cl1 background line (RMgm-7). Mature schizonts were intravenously (IV) administered to naïve TO mice. Ring stage parasites were isolated at 4 hpi and mature trophozoites and gametocytes were removed by passing through a MACS LD column (Miltenyi Biotec). Infected erythrocytes were incubated in −SerM medium, −SerM medium supplemented with 20 μM LysoPC (−SerM/LysoPC), or serum-complemented medium (+SerM) for 20 hours. Mature schizont stage parasites were then isolated on a 55% Nycodenz (Axis-Shield POC)/RPMI gradient and injected intravenously into 2 or 3 naïve mice. GFP-expressing cells were examined by flow cytometry at 16 hpi to calculate parasitemia, while cells expressing both RFP and GFP (gametocytes) were assessed at 21 hpi. Gametocytemia was calculated as [(RFP^+^ and GFP^+^ cells)/GFP^+^ cells]^∗^100.

#### Reticulocyte isolation

Reticulocytes were isolated as described (Clark et al., 2014, Sorette et al., 1992). Briefly, cells were enriched from leukocyte-depleted blood in a two-step process. First, 500 mL of leukodepleted blood was aliquoted into 50 mL conicals and erythrocytes were spun for 1 hour at 4000 g. Plasma was removed and the top 20% of packed cells was carefully collected. Cells from this first enrichment step were then resuspended at a hematocrit of 50% in plasma and subsequently layered at a 1:1 ratio onto a 1.080 g/mL KCl high Percoll (Sigma-Aldrich) density gradient and spun for 25 minutes at 1200 g. The reticulocyte-enriched interphase was collected and washed twice with 10x volume of RPMI to remove residual Percoll. Enriched reticulocytes were subsequently stored at a minimum hematocrit of 10% in RPMI medium containing 0.5% Albumax, 25 mM HEPES, and 24 mM sodium bicarbonate. High purity isolation of CD71-positive reticulocytes was performed according to standard MACS procedures for microbead isolation of CD71+ cells, as suggested by the manufacturer (Miltenyi Biotec).

#### Transcriptional profiling

*P. falciparum* NF54 and Pf2004/164tdTom parasite cultures were synchronized to a 4-hour time window using consecutive sorbitol (Sigma-Aldrich) treatments and exposed to conditions that induce (−SerM) or prevent sexual commitment (−SerM/LysoPC (20 μM LysoPC); +SerM) at 30±2 hpi. Total RNA was collected in 1 mL Trizol (Invitrogen) from approximately 5x10^8^ parasites per condition at this time point and in 4-hour intervals thereafter and stored at -80°C. Prior to Trizol treatment, erythrocytes were lysed in 0.015% saponin (room temperature, 5 min, from Sigma-Aldrich) and parasites were pelleted (3000 g, 5 min) and washed three times in 10 mL PBS at room temperature. RNA was isolated from 500 μL Trizol solution by three extraction steps (1x using 500 μL phenol:chloroform:isoamyl alcohol (25:24:1); 2x using 100 μL chloroform). After vigorous shaking for 30 seconds, phases were separated by centrifugation (12000 g, 10 min, 4°C). RNA was purified from 200 μL of the aqueous phase using an RNeasy micro kit (Qiagen) and corresponding protocol. RNA was eluted in 32 μL DEPC-treated water, quantified by Qubit (Life Technologies) and stored at -80°C.

0.5 μg - 1.0 μg total RNA of each sample was used for library preparation. Libraries were prepared by using TruSeq Stranded mRNA kit (Illumina). Library quantification was conducted by qPCR and TapeStation (Agilent Technologies) measurements. Libraries were subsequently pooled and sequenced using 300 cycle V2 MiSeq reagent kit (Illumina).

#### Imaging of LysoPC in live cells

Prior to imaging, *P. falciparum* early trophozoite-infected RBCs were incubated in either −SerM (inducing), −SerM conditions supplemented with 20 μM LysoPC (−SerM/LysoPC; non-inducing), or serum-complemented medium (+SerM; non-inducing). Parasites were allowed to develop until the trophozoite stage (26±2 hpi), late trophozoite stage (30±2 hpi), early schizont (36±2 hpi), late schizont (42±2 hpi), or ring stage (4±2 hpi).

Parasites were processed as described by Grüring *et al.* (Grüring et al., 2011). Briefly, cells from −SerM, −SerM/LysoPC, or +SerM culture were arrested on the glass bottom of a sterile, concanavalin A (ConA, Sigma-Aldrich)-coated dish. ConA was dissolved at a concentration of 0.5 mg per mL in H_2_O, and 200 μL were distributed uniformly on the glass surface of a 27 mm dish (Scientific Laboratory Supplies). ConA was added to the dish for 30 minutes at 37°C. It was then washed off twice, using 1x PBS, after which the culture was re-suspended in PBS, added to the glass bottom of the dish, and allowed to settle for 20 minutes at 37°C. Thereafter, non-bound cells were washed off using PBS, leaving a monolayer in the glass bottom, and 2 mL of pre-warmed PBS were added to the dish for imaging. Cells were viewed using a Zeiss Observer Z1 spinning disc confocal microscope equipped with an incubation chamber, a Yokogawa CSU-X1 filter wheel and spinning disc unit, a Photometrics Evolve 512 delta EM-CCD camera and four laser lines: 405, 488, 561, and 642 nm, and an α-Plan-Apochromat 100x 1.46 NA DIC VIS immersion oil lens.

Immediately prior to imaging, cells were incubated in either 20 μM TopFluor LysoPC (1-(dipyrrometheneboron difluoride) undecanoyl-2-hydroxy-sn-glycero-3-phosphocholine); in 20 μM TopFluor phosphatidylcholine (PC) (1-palmitoyl-2-[11-(dipyrrometheneboron difluoride) undecanoyl]-sn-glycero-3-phosphocholine); or in 20 μM NBD phosphatidic acid (PA) (1-palmitoyl-2-{12-[(7-nitro-2-1,3-benzoxadiazol-4-yl] amino} dodecanoyl)-sn-glycero-3-phosphate (ammonium salt)) (all from Avanti Polar Lipids). Lipids were dissolved in methanol and stored as stocks at a concentration of 1 mM. For use, the lipid solution was added to a dish and the methanol was allowed to evaporate under sterile conditions. The lipids were then re-suspended in PBS to a concentration of 20 μM prior to addition to the iRBCs. For ER colocalization and nuclear staining, ER tracker red (Bodipy TR glibenclamide, from Invitrogen) and Hoechst 33342 (ThermoScientific) were used, respectively. Cells were viewed using 40 nm (Hoechst), 488 nm (TopFluor) or 561 nm (ER-tracker and dsRed) laser lines.

Image collection parameters for localization at different stages were 512 x 512 d.p.i, 50-72 z-stacks (0.2 μm step size), a zoom level of 1-2 and laser levels of 1-5% for 488 nm, 1-10% for 561 nm, and 10% for 405 nm. Image collection parameters for time-lapse imaging of LysoPC uptake and co-localization with ER-tracker were 512 x 512 d.p.i, at an interval of 1 s per frame. Image acquisition was initiated (time zero) prior to the addition of the labeled dye. Without interruption of image acquisition, 20 s after acquisition of the first image, the dissolved labeled dye was added to the culture dish, and image acquisition continued to visualize uptake of the dye and distribution within the infected erythrocyte.

To avoid crosstalk between channels images were collected in line sequential mode with z-increments of 0.19 μm. Images were acquired using the Zen 2012 (Zeiss) software. Images were analyzed with Fiji (Schindelin et al., 2012) and Imaris (Bitplane) software. 100 images were quantified and analyzed per condition and time point.

#### Quantification of merozoite numbers

*P. falciparum* trophozoite-infected erythrocytes were incubated in either −SerM medium (inducing), −SerM medium supplemented with 20 μM LysoPC (−SerM/LysoPC; non-inducing), or serum-complemented media (+SerM; non-inducing). Following a 4-6 hour incubation period, parasites were incubated in +SerM, and allowed to develop until 32±2 hpi. Parasites were then immobilized in a glass-bottom dish as described above, stained with Hoechst 33342, and 50-72 z-stacks (0.2 μm step size) obtained using bright field and the 405 nm laser line, to determine the number of nuclei in every infected erythrocyte. This procedure was repeated bi-hourly for a total of 18 hours per condition, and 50-100 images quantified per time point and condition.

Synchronized and newly invaded *P. berghei* ring stage parasites were isolated as described earlier. Infected erythrocytes were incubated in −SerM conditions, −SerM supplemented with 20 μM LysoPC (−SerM/LysoPC), or serum-complemented medium (+SerM). At the indicated time points, infected erythrocytes were fixed in 4% paraformaldehyde/0.075% glutaraldehyde in PBS for 10 minutes, washed twice in PBS, smeared on glass slides and mounted in Vectashield containing DAPI (Vectorlabs). Z-stacks of 4-5 μm (0.1 μm step) were acquired on a DeltaVision epifluorescence microscope (Applied Precision) using the 405 nm and bright field filter sets. Images were deconvoluted using SoftWoRx to count number of merozoites.

#### Calculation of LysoPC levels from published data

We compared our *P. berghei*-infected mouse LysoPC quantification with published levels of LysoPC in parasite- and bacteria-infected humans. In the case of bacterial sepsis (Drobnik et al., 2003) and *P. aeruginosa*-infected cystic fibrosis patients (Ollero et al., 2011), LysoPC concentrations from infected and uninfected hosts were explicitly reported. In these cases, the reported values were directly plotted in Figure 6A. In the case of *P. falciparum* infection (Lakshmanan et al., 2012, Orikiiriza et al., 2017) and *T. brucei* infection (Lamour et al., 2015), only relative quantities of LysoPC in infected versus uninfected hosts were reported. To calculate absolute LysoPC concentrations from these data, we set the uninfected LysoPC concentrations to the levels published for healthy individuals (Psychogios et al., 2011), and calculated LysoPC concentrations of the infected individuals based on the reported relative quantification data. The uninfected data and calculated infected data were plotted in Figure 6A.

#### Generation of CRISPR/Cas9 and donor plasmids

Using the previously published transfection vectors pUF1_Cas9 and pL-6_eGFP (Ghorbal et al., 2014) as templates, we generated a new CRISPR/Cas9 plasmid termed p_gC that contains expression cassettes for the *Streptococcus pyogenes* Cas9 enzyme and the single guide RNA (sgRNA). In addition, we inserted into p_gC a HindIII-SalI-EcoRI-BamHI multi-cloning site for insertion of a drug resistance cassette and replaced the original BtgZI recognition site in the U6 sgRNA expression cassette with a BsaI site for directional insertion of the sgRNA target sequence. We then inserted a h*dhfr* resistance cassette into BamHI-digested p_gC to obtain the Cas9/sgRNA mother plasmid pH_gC. To engineer marker-free parasites expressing AP2-G-GFP from the endogenous locus (PF3D7_1222600) we generated transfection vectors pH_gC-*ap2g-3’* (Cas9/sgRNA suicide plasmid) and pD_*ap2g-gfp* (donor plasmid) (Supplementary Figure S4). To generate pH_gC-*ap2g-3’*, two complementary oligonucleotides (sgt_*ap2g3’*_F, sgt_*ap2g3’*_R) encoding the sgRNA target sequence sgt_*ap2-g*-3’ and appropriate single-stranded overhangs were annealed and inserted into the sgRNA expression cassette using *Bsa*I-digested pH_gC and T4 DNA ligase. The sgRNA target sequence sgt_*ap2g3’* (AGTTATAGGGAATATTCAAA) is positioned 60bp downstream of the *ap2-g* coding sequence and was identified using CHOPCHOP (Labun et al., 2016). The donor plasmid pD_*ap2g-gfp* was generated using Gibson assembly joining four PCR fragments encoding (1) the plasmid backbone amplified from pUC19 using primers PCRA_F and PCRA_R, (2) the *ap2-g* 5’ homology box amplified from 3D7 gDNA using primers PCRB_F and PCRB_R, (3) the *gfp* coding sequence (plus N-terminal GSAG linker) amplified from pH-GFP (Brancucci et al., 2014) using primers PCRC_F and PCRC_R, and (4) the *ap2-g* 3’ homology box amplified from 3D7 gDNA using primers PCRD_F and PCRD_R. All oligonucleotide sequences are provided in Supplementary Table S1.

#### Parasite transfection and selection

NF54 ring stage parasites were transfected simultaneously with 50 μg pH_gC-*ap2g-3’* (Cas9/sgRNA suicide plasmid) and 50 μg pD_*ap2g-gfp* (donor plasmid) using electroporation conditions as described (Voss et al., 2006). To select for parasites carrying the tagged *ap2-g* locus (NF54/AP2-G^GFP^), transfected parasites were grown in presence of 4 nM WR99210 for the first six days and then in absence of drug pressure until a stably propagating parasite population was established (approximately four weeks post-transfection). Successful tagging of the *ap2-g* locus in the NF54/AP2-G^GFP^ population was confirmed by PCR on gDNA (Figure S4). All oligonucleotide sequences are provided in Table S1.

### Quantification and Statistical Analysis

Except for RNA-seq data analysis (see below), statistical details can be found in the figure and figure legends.

#### RNA-seq experiments and data analysis

RNA-seq reads from each sample were aligned to the *P. falciparum* reference genome (PlasmoDB version 28). A maximum of one mismatch per read was allowed. The mapped reads from TopHat (Kim et al., 2013) were used to assemble known transcripts from the reference and their abundances were estimated using Cufflinks (Trapnell et al., 2010). The expression level of each gene was normalized as FPKM (fragments per kilobase of exon per million mapped reads). We developed an induction score (*IS*) to measure transcriptional responses of genes to different culture conditions. For two strains (Sϵ *NF54, Pf2004*) in different conditions (Cϵ +*SerM,* −*SerM* and −*SerM/LysoPC*), the transcription level (*Exp*) of each gene *i* in 4 time points (Tϵ *TP1, TP2, TP3 and TP4*) can be represented as $Exp_{i,T}^{S,C}$. For each time points, we first paired condition $C_{1}$ ($\;C_{1}\epsilon$ -*SerM* ) with $C_{2}$ ($\;C_{2}\epsilon$ +*SerM,* −*SerM/LysoPC* ) and represented as $Exp_{i,T}^{S,C_{1}}$ vs. $Exp_{i,T}^{S,C_{2}}$ . 8 (4 time points × 2non-inducing) pairs can be obtained and the *IS* can be represented as

$$IS=log2\left[\sum_{T,C_{2}}\frac{Exp_{i,T}^{S,C_{2}}+1}{Exp_{i,T}^{S,C_{1}}+1}\right]$$

To identify deregulated genes, the group of non-induced parasites can be represented as vector

$\left(Exp_{i,\;TP1}^{NF54,+SerM},\ldots ,Exp_{i,\;TP4}^{Pf2004,+SerM},\;Exp_{i,\;TP1}^{NF54,-SerM/LysoPC},\ldots ,Exp_{i,\;TP4}^{Pf2004,\;-SerM/LysoPC}\right)$, while the induction group is

$\left(Exp_{i,\;TP1}^{NF54,-SerM},\ldots ,Exp_{i,\;TP4}^{Pf2004,-SerM},\;Exp_{i,\;TP1}^{NF54,-SerM},\ldots ,Exp_{i,\;TP4}^{Pf2004,\;-SerM}\right)$. Each vector has 16 elements (8 × 2 strains) and Paired Wilcoxon test and BH p value adjustment were implemented. Genes with an adjusted p value of less than 0.05 were identified as significantly differentially regulated. Gene Ontology (GO) annotations were obtained from PlasmoDB version 28. The Barnard test (Barnard, 1947) was used to test enrichment of specific GO terms in each gene group against the genomic background.

### Data and Software Availability

The accession number for the transcriptional data reported in this paper is GEO: GSE104114.

## Author Contributions

N.M.B.B. and J.P.G. designed and performed experiments, analyzed and interpreted data, prepared illustrations, and wrote the manuscript. M.D.N. and N.M.B.B. performed and analyzed microscopy experiments. A.B.S., M.-C.L., and N.M.B.B. performed EV and BSA experiments. N.P. and M.D.N. performed and analyzed *P. berghei* experiments. M.D.N. and J.P.G. performed *in vivo* extraction and LysoPC analysis from *P.berghei*-infected and non-infected mouse tissues. M.Z. processed RNA samples and performed MiSeq sequencing. C.W. and S.R.A. analyzed RNA-seq data. M.A.C. performed reticulocyte purifications and provided experimental advice, supervised by M.T.D. D.R. supported RNA-sample isolation and processing. E.H., S.D.B., and I.N. generated the NF54/AP2-G^GFP^ line, and T.S.V. supervised these experiments. M.-C.L. assayed the role of BSA in LysoPC activity. C.G. transfected Pf2004 parasites with the 164tdTom reporter plasmid. E.G.-D. synthesized *OMe*LysoPC. B.R.-R. and A.C.-G. synthesized choline-kinase inhibitor compound BR23. A.D. and S.B. optimized −SerM composition, and D.F.W. supervised these experiments. J.H.A. and R.H.Y.J. supervised RNA-seq data generation and data interpretation. A.P.W. supervised mouse model work. M.M. and J.C. designed the study, interpreted data, and supervised the study. N.M.B.B., J.P.G., J.C., and M.M. wrote the manuscript with input from all co-authors.

## Supplemental Information

Table S1. Oligonucleotides Used in This Study, Related to STAR Methods

Dataset S1. LC-MS Retention Times and Masses for Standards, Related to Figure 3 Data from metabolic labeling experiments using ^13^C palmitate LysoPC, ^2^H choline LysoPC and ^2^H choline (.xlsx file).

Dataset S2. Related to Figure 4 RNA-seq data comparing parasite transcription in presence and in absence of LysoPC (.xlsx file).
