# Supplementary material for: Lysophosphatidylcholine Regulates Sexual Stage Differentiation in the Human Malaria Parasite Plasmodium falciparum
Source: Cell. 2017 Dec 14;171(7):1532–1544.e15. doi: 10.1016/j.cell.2017.10.020 (PMC5733390; doi:10.1016/j.cell.2017.10.020)
Supplement: Table S1. Oligonucleotides Used in This Study, Related to STAR Methods [file mmc1.pdf]

Cell, Volume *171*

Supplemental Information

Lysophosphatidylcholine Regulates Sexual Stage Differentiation in the Human Malaria Parasite *Plasmodium falciparum*

Nicolas M.B. Brancucci, Joseph P. Gerdt, ChengQi Wang, Mariana De Niz, Nisha Philip, Swamy R. Adapa, Min Zhang, Eva Hitz, Igor Niederwieser, Sylwia D. Boltryk, Marie-Claude Laffitte, Martha A. Clark, Christof Grüring, Deepali Ravel, Alexandra Blancke Soares, Allison Demas, Selina Bopp, Belén Rubio-Ruiz, Ana Conejo-Garcia, Dyann F. Wirth, Edyta Gendaszewska-Darmach, Manoj T. Duraisingh, John H. Adams, Till S. Voss, Andrew P. Waters, Rays H.Y. Jiang, Jon Clardy, and Matthias Marti

Supplementary Table S1. Related to STAR Methods Details sections “Generation of CRISPR9/Cas9 and donor plasmids” and “Parasite transfection and selection”.

Oligonucleotides used in this study.

**Name**	**Direction**	**Sequence (5’-3’)**
sgt_*ap2g3’*_F	F	TATTAGTTATAGGGAATATTCAAA
sgt_*ap2g3’*_R	R	AAACTTTGAATATTCCCTATAACT
PCRA_F	F	CTGGCGTAATAGCGAAGAGG
PCRA_R	R	CATTAATGAATCGGCCAACG
PCRB_F	F	CGTTGGCCGATTCATTAATGGATGCTACCATAACATTTAATAACC
PCRB_R	R	ACTCATACCTGCGGATCCAATATTCCTGTTGTTTCCCC
PCRC_F	F	ATTGGATCCGCAGGTATGAGTAAAGGAGAAGAACTTTTC
PCRC_R	R	ATAGGGAATATTCAAAATCATTTGTATAGTTCATCCATG
PCRD_F	F	AATGATTTTGAATATTCCCTATAACTAAATG
PCRD_R	R	CCTCTTCGCTATTACGCCAGCTGTGTGTTGAGAAATTTAAAG
ap2F	F	GGTAATGATGGTGCATTGAG
ap2R	R	GTACAACAAAAACTGAACTC
gfpF	F	GGTTATGTACAGGAAAGAAC
gfpR	R	GTGTGAGTTATAGTTGTATTCC
